# Proteomics and metabolomics analyses of camptothecin-producing *Aspergillus terreus* reveal the integration of PH domain-containing proteins and peptidylprolyl cis/trans isomerase in restoring the camptothecin biosynthesis

**DOI:** 10.1128/spectrum.02281-23

**Published:** 2023-10-19

**Authors:** Amgad M. Rady, Ashraf S. A. El-Sayed, Ashraf F. El-Baz, Ghada G. Abdel-Fattah, Sameh Magdeldin, Eman Ahmed, Aya Osama, Sameh E. Hassanein, Hend Saed, Marwa Yassin

**Affiliations:** 1 Enzymology and Fungal Biotechnology Lab (EFBL), Botany and Microbiology Department, Faculty of Science, Zagazig University, Zagazig, Egypt; 2 Faculty of Biotechnology, October University for Modern Sciences and Arts, Giza, Egypt; 3 Genetic Engineering and Biotechnology Research Institute, University of Sadat City, Sadat City, Egypt; 4 Botany Department, Faculty of Science, Mansoura University, Mansoura, Egypt; 5 Proteomics and Metabolomics Research Program, Department of Basic Research, Children’s Cancer Hospital, Cairo, Egypt; 6 Department of Physiology, Faculty of Veterinary Medicine, Suez Canal University, Ismailia, Egypt; 7 Department of Pharmacology, Faculty of Veterinary Medicine, Suez Canal University, Ismailia, Egypt; 8 Agricultural Genetic Engineering Research Institute (AGERI), Agriculture Research Center, Cairo, Egypt; 9 Microbiology Department, Faculty of Veterinary Medicine, Zagazig University, Zagazig, Egypt; Geisel School of Medicine at Dartmouth, Lebanon, New Hampshire, USA

**Keywords:** camptothecin, *Aspergillus terreus*, proteomics analysis, metabolomics analysis

## Abstract

Attenuating the expression of fungal camptothecin biosynthetic genes with subculturing is the challenge that halts their further implementation. The camptothecin productivity of the subcultured *Aspergillus terreus* has been restored upon addition of *Ficus elastica* indigenous microbiome; however, the identity of triggering signals of *A. terreus* camptothecin biosynthesis remains ambiguous. In this study, differential proteomics and metabolomics analyses were implemented to unravel the differentially abundant proteins and metabolites associated with the weakening/restoration of the biosynthetic machinery of camptothecin by *A. terreus*. The functional proteins, namely, ribosomal proteins, ATP, metal ion, and GTP binding proteins, were abolished by the seventh culture of *A. terreus*; however, the expression of these proteins was completely restored upon addition of *F. elastica* microbiome. Among the proteins of highly altered abundance, Pleckstrin homology (PH) domain-containing protein, peptidylprolyl *cis*/*trans* isomerase, 60S ribosomal protein, and So-Cu domain-containing proteins were significantly decreased with subculturing of *A. terreus* and strikingly restored upon addition of *F. elastica* microbiome. The metabolites 5,7-dihydroxy-2-(4-hydroxyphenyl)-3,6-dimethoxy-4H-chromen and glutaric acid of *A. terreus* were significantly decreased with subculturing and completely restored upon addition of *F. elastica* microbiome. The most differentially abundant metabolites were involved in glycolysis, TCA cycle, mevalonate pathway, terpenoids and shikimate synthesis, and ultimately with camptothecin biosynthesis. Thus, overexpression of PH domain-containing protein and peptidylprolyl *cis*/*trans* isomerase could be a new avenue for a metabolically stable camptothecin producing *A. terreus*.

**IMPORTANCE:**

Decreasing the camptothecin productivity by fungi with storage and subculturing is the challenge that halts their further implementation to be an industrial platform for camptothecin (CPT) production. The highest differentially abundant proteins were Pleckstrin homology (PH) domain-containing proteins and Peptidyl-prolyl *cis*/*trans* isomerase that fluctuated with the subculturing of *A. terreus* with a remarkable relation to CPT biosynthesis and restored with addition of *F. elastica* microbiome.

## INTRODUCTION

Camptothecin (CPT) is a pentacyclic pyrroloquinoline alkaloid, isolated from *Camptotheca acuminata* in China and India (1). CPT has been recognized as a potent antiproliferative agent toward various tumor cells, due to its unique affinity for binding with DNA Topoisomerase I of tumor cells, inducing protein-DNA breakage (2). The blocking of Topoisomerase I activity by CPT stops the relaxation of the DNA supercoiling during the successive multiplications of tumor cells (3, 4). The Topoisomerase I creates a nick in the single DNA strand releasing the supercoils generated from the multiple replications of tumor cells, via an ester linkage with the 3′end of nicked DNA (3). CPT is a monoterpenoid indole alkaloid derived from condensation of secologanin and tryptamine “decarboxylated tryptophan” to form strictosidine by strictosidine synthase (STR) (5). Tryptamine is derived from decarboxylation of tryptophan by tryptophan decarboxylase (TDC), while secologanin is derived from the terpenes biosynthetic pathway (6). The committed steps of CPT biosynthesis in plants and fungi are controlled by some rate-limiting enzymes such as geraniol synthase (GES), secologanin synthetase (SLS), strictosidine synthase, strictosidine β-glucosidase (SGD), and tryptophan decarboxylase (7).

Camptothecin is one the most common commercial anticancer drugs (8); however, the productivity of this compound is the challenge that is halting their clinical applications (9 – 12). *Camptotheca acuminata* that ecologically inhabits Asian areas “China and India” was the main CPT source; however, the tiny yield with the heavy demand of this compound resulted in destructive harvesting of this plant (10, 13). Ecologically, *C. acuminata* has been scarcely inhabiting the Middle East, which makes extra difficulties in obtaining this drug. Additionally, the steric complexity, bulky compounds, and difficulty of extraction are the extra challenges for the acquisition of CPT from these plants (12). Intriguingly, the metabolic potency of fungi for biosynthesis of CPT elevates the prospective industrial production of CPT (9, 14 – 18) (15). The rationality of fungi for the commercial production of bioactive metabolites elaborates from their fast growth rates, obtainability of bulk biomass, independence on the environmental conditions, and feasibility of metabolic and molecular manipulation (19 – 24). Camptothecin has been firstly isolated and chemically resolved from *Entrophospora infrequens*, followed by numerous fungal endophytes (21, 25). *Aspergillus terreus*, an endophyte of *Ficus elastica* (9, 15), *Cestrum parqui* (15), *Cinnamomum camphora* (26), *Aspergillus flavus*, “endophyte of *Astragalus* sp.” (17), and *Penicillium chrysogenum* (20) have been reported as a potent CPT producers. However, the suppression of CPT productivity with fungal storage and subculturing are the challenges that limits their further implementation (9, 14 – 16, 20, 27, 28).

*Aspergillus terreus*, an endophyte *F. elastica*, has been recognized frequently with its relative stability and sustainability as revealed from our studies (9, 15, 17, 20, 26), comparing with other CPT-producing fungi. The yield of CPT by *A. terreus* was decreased sequentially with storage and multiple subculturing (9). The biosynthetic machinery of CPT by *A. terreus* was completely restored upon addition of the entire microbiome of the host plants, “*F. elastica*,” confirming the secretion of specific chemical signals from the CPT- and/or non-CPT-producing endogenous microbes that triggers the CPT biosynthetic gene cluster of *A. terreus* (9, 15, 17, 20, 26). However, the mechanism of attenuation of CPT production by *A. terreus* with subculturing and storage and restoring of their biosynthesis upon addition of the plant microbiome remains equivocal. Thus, the objective of this study was to unravel to the metabolic machinery associated with attenuation and/or restore the biosynthetic machineries of CPT by *A. terreus* in response to addition of indigenous microbiome of *F. elastica*, at the proteomics and metabolomics levels.

## MATERIALS AND METHODS

### *Aspergillus terreus* growth conditions, CPT extraction, and HPLC quantification

The potent CPT-producing *Aspergillus terreus* EFBL22 (MW040820 and AUMC1391), an endophyte of *Ficus elastica*, has been used for further CPT biosynthetic stability studies, as in an extension to our previous study (9). The fungal isolate was grown on the nutritionally optimized media of the Plackett-Burman design, under standard growth conditions (9). A plug of the fungal isolate of 6-day-old cultures was inoculated into 50 mL of medium/250-mL Erlenmeyer flask and incubated for 14 days at 30°C. Triplicates of the fungal cultures were made. The cultures were filtered by sterile cheesecloth, and the filtrates were centrifuged at 5,000 rpm for 10 min, followed by downstream CPT extraction by CHCl_3_:MeOH (4:1) (1, 18, 22). The extract was fractionated by thin layer chromatography (TLC) plates with the solvent system chloroform:methanol (9:1, vol/vol) (22), the plates were visualized at λ_254_ nm, and the spots gave the same color and relative mobility of the authentic one (Cat. #7689-0 3-4), were scraped off, and dissolved in methanol (29, 30). The purity of the extracted CPT was determined by high performance liquid chromatography (HPLC) (Chromass, 9110+ Quaternary Pump, Korea), RP-C18 column (Cat. #959963-902) with phase methanol/water (60:40 vol/vol) at a flow rate of 1.0 mL/min for 20 min, scanned by a photodiode array detector. The identity and concentration of the sample were authenticated from the retention time and peak area of the standard one at λ_360_ nm.

### Camptothecin biosynthetic stability with *A. terreus* subculturing and in response to amendment with *F. elastica* microbiome

The productivity of CPT by *A. terreus* responsive to the successive subculturing was assessed. The zero axenic culture of *A. terreus* was subcultured to the 12 generations by the hyphal tip method, with an interval lifespan of 10 days, as a slope cultures on PDA (18, 24, 31, 32). *A. terreus* was gown on the potato dextrose broth media and incubated under the standard conditions, and the CPT was eluted and assessed by TLC and HPLC. The influence of *F. elastica* surface-sterilized leaves on *A. terreus* CPT productivity was assessed, the sections of leaves were amended to 5-day-old culture of *A. terreus* at different concentrations (0.1%–10.0%) (9, 15). Negative controls of the sterilized *F. elastica* leaves inoculated to blank PDB media were used. The cultures were incubated, and CPT was extracted and quantified as described above.

### Molecular expression analyses of the rate-limiting CPT biosynthetic genes by RT-PCR

Mining of the CPT rate-limiting biosynthetic genes was used as a molecular marker for assessing the biosynthetic machinery of CPT in the fungal genome (9, 15, 17, 20, 26). The committed biosynthetic steps of CPT are controlled by secologanin synthetase and tryptophan decarboxylase (10, 33, 34). The primers sets of *sls* 5′-TGCTCAACTGGGCGTATTT-3′, 5′-CCTCATCCTGTTGTTCCTCTTAG-3′, and *tdc* 5′-CAAGCCCATCGTATGGTAGATT-3′, 5′-GATTCGTAGTGAGTGCCCTTAG-3′ were used. RNA was extracted by RNeasy Kit (QIAGEN, USA), and the cDNA was synthesized by the SuperScript III First-Strand Synthesis Kit with oligo-dT primes (35, 36). The reaction contains 10 µL of 2× PCR Master Mixture (Cat. #25027), 2 µL cDNA, and primers (10 pmol), in 20 µL volume. The PCR was programmed to initial denaturation 94°C for 4 min, 35 cycles at 94°C for 20 s, 51°C for 30 s, 72°C for 30 s, and final extension 5 min at 72°C. The relative expression of the genes was determined by ImageJ software package, normalizing to *actaA* gene of *A. terreus*.

### Proteomics analyses

#### Sample preparation, trypsinization, and cation exchange fractionation

The effect of the amendment of *A. terreus* cultures with the leaves of *F. elastica* on the molecular biosynthetic machinery of CPT was assessed from the differential proteomic analyses. The zero-culture *A. terreus*, seventh cultures of *A. terreus*, and seventh cultures amended with 1% surface-sterilized leaves of *F. elastica* were incubated at standard conditions as mentioned above. The cultures were filtered, and the intracellular proteins were extracted for proteomic analysis. The fungal biomass were pulverized in liquid nitrogen, dissolved in extraction buffer (formic acid and 100% acetonitrile), for 10 min in ice, and vortexed; then, 200 µL of 8 M urea with 20 µL of protease inhibitor cocktail was added and incubated for 15 min in ice. The samples were centrifuged at 10,000 rpm for 30 min; the protein contents were measured by Folin’s solution (35, 37). Forty micrograms of extracted proteins was reduced by 200 mM DTT, incubated at 30°C for 30 min, followed by 2 µL of 1 M iodoacetamide, and incubated for 1 h in the dark, with the addition of 100 µL of 0.1 M Tris-HCl (pH 8.5), to reduce the urea ratio before trypsinization (38). The samples were digested with 1 µg procaine-trypsin (40:1), incubated for 12 h at 37°C with shaking, acidified to pH 2–3 with 100% formic acid, and centrifuged at 10,000 rpm for 30 min, and the peptide mixture was fractionated with Stage Tip (PierceC18 Spin Tips, Cat. #84850) (39). The peptides were activated by 15 µL methanol and 15 µL solution B (0.2% formic acid and 80% acetonitrile), pre-equilibrated by 15 µL solution A (0.2% formic acid), washed with 15 µL of solution A.

### LC-MS/MS analysis, peptide identification, and mass spectrometry

The analysis of the proteins was conducted by the NanoLC system with Eksigent NanoLC 400 autosampler and Ekspert NanoLC pump coupled to the LC-QTOF system of Sciex Triple TOF 5600+. The fractions were enriched by nano-trap column, and the trapped peptides were separated for 55 min with trapping cartridge Chrom XP C18CL 5 µm at flow rate 10 µL/min for 3 min. The mobile phase consists of eluent A (0.1% vol/vol, formic acid in H_2_O) and eluent B (0.1% vol/vol, formic acid in 90/10 acetonitrile). The analysis was performed in positive ion mode, the scan range was 400–1,250 m/z, the MS2 range was 170–1,500 m/z, and the peptides were identified with Swiss-Prot of *A. terreus* (http://www.ncbi.nlm.nih.gov/genome).

### Peptide identification, relative quantification, and bioinformatics analysis

The retrieved sequences were non-redundantly annotated by Protein Pilot (v4.5) software package. The data were normalized, the differential analyses were done, and the *P*-values were calculated. The expression change has been considered as a significant if the protein fold change ≥ 1.2 or ≤0.8 with a *P* ≤ 0.05. Annotation of the differentially abundant proteins was performed by the Gene Ontology (GO) enrichment tool in fungi (40, 41); the GO biological process was visualized by REVIGO outputs with R-Studio (v3.3.0) (41, 42). Venn diagrams of the differentially abundant proteins were made by Venny (v2.1). The enrichment analyses were performed by the metabolic pathway analyses tool in Fungal DB with Kyoto Encyclopedia of Genes and Genomes (KEGG) (43, 44). The protein-protein interactions were analyzed with STRING (v10.5) (43).

### Metabolomics analyses

#### Sample preparation

The lyophilized fungal biomass (50 mg) was dispensed in 1 mL solution (water:methanol:acetonitrile, 2:1:1), vortexed for 2 min, and then sonicated at 20–30 kHz for 10 min. One milliliter of the reconstitution solvent was added and centrifuged at 10,000 rpm for 5 min; 10 µL was injected at concentration 1 µg/µL. Blank samples are underwent liquid chromatography with tandem mass spectrometry (LC-MS/MS) analysis for quality assurance of the experiment. The molecules were separated on a Axion AC System (Kyoto, Japan) with XSelect HSS T3 (2.5 µm, 2.1 × 150 mm) column maintained at 40°C and flow rate 300 µL/min. The mobile phase consists of solution A (5 mM ammonium formate in 1% methanol of pH 3.0), solution B (acetonitrile), and solution C (5 mM ammonium formate in 1% methanol with pH 8.0). The gradient elution was performed with the following: 0–20 min, 10% B; 21–25 min, 90% B; 25.01–28 min, 10% B; and then 90% B for equilibration of the column (45, 46). Mass spectrometry was performed on a Triple TOFTM 5600+ system quadrupole-TOF mass spectrometer. The voltage floating (ISVF) and voltages were +4,500 and +80 V in positive mode and −4,500 and −80 V in negative mode, respectively. Batches of MS and MS/MS data collection were created using Analyst TF 1.7.1. High-resolution survey spectra from 50 to 1,100 m/z and the mass spectrometer were operated in a pattern where a 50-ms survey scan was detected (47).

### KEGG annotation and metabolic pathway analysis

The metabolites of *A. terreus* were annotated and identified using KEGG database by performing the metabolic pathway enrichment analysis of the differential metabolites in response to the amendment with *F. elastica* microbiome. The structure information and mass fragments were considered for further validation of the metabolite identities. The KEGG pathway online mapper was used to shows the differential metabolic pathways (44).

### Statistical analysis

Biological triplicates of the experiments were conducted, and the results were represented by means ± SD. The statistical analysis was assessed with one-way ANOVA (analysis of variance, SPSS software v.18), and the means were compared with Duncan’s test at 0.05 level.

## RESULTS

### Biosynthetic stability of camptothecin by *Aspergillus terreus* with the fungal subculturing

**Fig 1:**
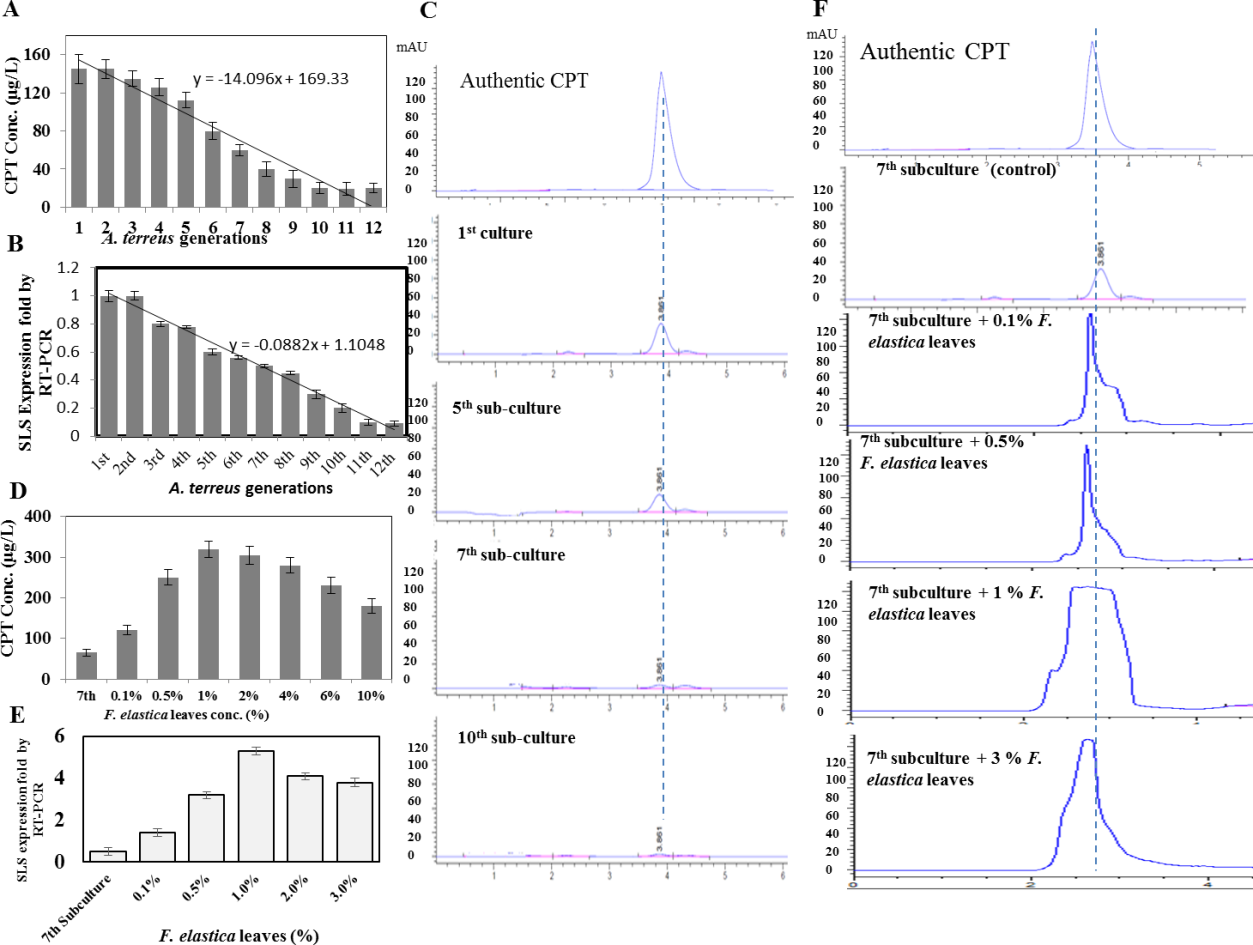
Productivity of CPT by *A. terreus* in response to successive subculturing and addition of with *F. elastica* leaves. The fungal isolate was sequentially subcultured till the 12th generation; the CPT productivity and the expression of secologanin synthase (*sls*) by the RT-PCR were determined. (**A**) The yield of CPT by the subcultures of *A. terreus*. (**B**) The expression of *sls* gene with the fungal subcultures. (**C**) HPLC chromatograms of the CPT eluted from the silica gel of TLC plates for the representative subcultures of *A. terreus*; the retention time of authentic CPT was 3.8 min. The seventh cultural generation of *A. terreus* was amended with different concentrations of surface-sterilized leaves of *F. elastica* (0.1%–3.0%). After incubation, CPT was extracted and quantified. Negative control cultures without *A. terreus* was used. (**D**) The CPT yield of *A. terreus* in response to addition of *F. elastica* leaves. (**E**) Molecular expression of the sls gene of CPT biosynthesis. (**F**) HPLC chromatograms of CPT of the seventh *A. terreus* subcultures, amended with *F. elastica* leaves.

The biosynthetic potency of CPT by *A. terreus* responsive to the subculturing was assessed. The zero isolate was subcultured till the 12th generation as slope culture on PDA, with a 10-day life span of each fungal culture; then, the CPT productivity for each culture was assessed under standard conditions. Practically, the CPT yield by *A. terreus* was sequentially decreased with the subculturing, ensuring the weakening of the CPT productivity with the subculturing (Fig. 1A and B). Camptothecin productivity by the first culture of *A. terreus* (145 µg/L) was attenuated by about 50% by the seventh cultural generation (65 µg/L) (Fig. 1C). A significant reduction to the yield of CPT by *A. terreus* by the 8th and 10th subcultures by approximately 75%–80%, comparing with the zero fungal culture, was observed. With the 12th cultural generation, the CPT productivity by *A. terreus* was reduced by >90%. Obviously, the relative metabolic stability of CPT was observed till the fifth cultural generation, assuming the physical stability of the inducing chemicals derived from the plant host associated with the fungal spores, these signals might be diluted with the subsequent generation (9). Interestingly, the conidial pigmentation and visual appearance of *A. terreus* on PDA media were observed with a noticeable fading from the rusty-yellow color of the original isolate to the bright-yellow coloring with the subsequent subculturing (data not shown). Also, the metabolic biosynthetic stability of CPT by *A. terreus* was evaluated based on the expression of secologanin synthetase. The expression fold of *sls* gene of *A. terreus* subcultures was assessed by semi-quantitative reverse transcription PCR (RT-PCR), normalizing to the *actaA* gene (48). From the results (Fig. 1B), a strong correlation has been observed between the expression of *sls* and the overall yield of CPT, with which the lowest expression of *sls* was assessed at the 10th to 12th subcultures. At the seventh subcultures, the expression of *sls* gene was suppressed by 50%, comparing to the zero cultures of *A. terreus*, authenticating the chromatographic detection of CPT. Thus, it could be deduced that the attenuation of the molecular machinery of CPT biosynthesis could be related to the upstream enzymatic machinery of the SLS enzyme that might be due to transcriptional factors. Strikingly, the decreasing of CPT productivity was observed as revealed from the TLC, HPLC being confirmed from the expression pattern of the *sls* gene of CPT biosynthesis.

### Restoring the biosynthetic potency of *A. terreus* CPT with the microbiome of *F. elastica*

Diminishing the CPT biosynthetic machinery is the common physiological criterion that halts the commercial applications of fungi for CPT production. Dependence of the CPT biosynthetic machinery of endophytic fungi on the biological signals derived from the entire microbiome of the plant host is the most acceptable hypothesis rationalizing this metabolic weakening (15, 17, 49). Thus, the sterilized leaves of *F. elastica* were added to 5-day-old culture of the seventh generation of *A. terreus*; after incubation, the CPT was extracted and quantified by HPLC (Fig. 1D and E). Practically, the CPT productivity by *A. terreus* was proportionally increased with the *F. elastica* leaf concentration. The CPT productivity by the seventh culture of *A. terreus* was not only completely restored but also increased by about twofold (320 µg/L) compared with the zero-culture *A. terreus* (145 µg/L) by the addition of 1% surface-sterilized parts of *F. elastica* leaves. Also, an obvious induction to the productivity of *A. terreus* CPT was noticed with higher of *F. elastica* leaves, with a slight reduction to the CPT yield at 3.0% leaf parts (Fig. 1F). So, retriggering of the attenuated CPT productivity of *A. terreus* could be due to a signal released from CPT-producing/or non-CPT-producing endophyte from the plant tissue, and with the intimate co-growth, the biosynthetic machinery of *A. terreus* CPT has been restored. Also, the restoration of productivity of *A. terreus* CPT was evaluated based on the expression of *sls* of secologanin synthesis, as a precursor of CPT. From the semi-quantitative RT-PCR (Fig. 1E), there is a noticeable induction to the molecular expression of the *sls* gene by about twofold and fivefold compared with the zero culture and seventh subculture of *A. terreus*, respectively, that is being completely matched with the metabolic analysis of CPT by the TLC and HPLC.

### Proteome profiling and Gene Ontology enrichment analyses of *A. terreus* in response to cocultivation with *F. elastica* microbiome

The metabolic biosynthetic machinery of CPT by *A. terreus* responsive to subculturing and amendment with microbiome of *F. elastica* was explored by the comparative proteomic analyses. From the CPT productivity profile, the seventh generation of *A. terreus* loss more than 50% of its initial CPT productivity, while, with the addition of 1% F. *elastica*-sterilized leave parts, the CPT productivity was completely restored and over-increased than the control cultures. The total intracellular proteins from the zero cultures, seventh culture of *A. terreus*, and seventh culture amended with 1% leaves of *F. elastica* were extracted for the proteomic analysis. Technical triplicates were used as independent LC-MS/MS sets. About 1,070 MS/MS spectra were generated (Table S1). The spectra were filtered with the universal false discovery rate (FDR), resulting in about 1,000 spectra, with an average of 500 distinct peptides identified from the protein-pilot coupled with NCBI and UniProt databases, identifying about 170 proteins/replica. The proteomics data were deposited on the massive repository http://massive.ucsd.edu/ProteoSAFe/status.jsp?task=2ac75562ddda42bba79512b3cf4c63b6.

**Fig 2:**
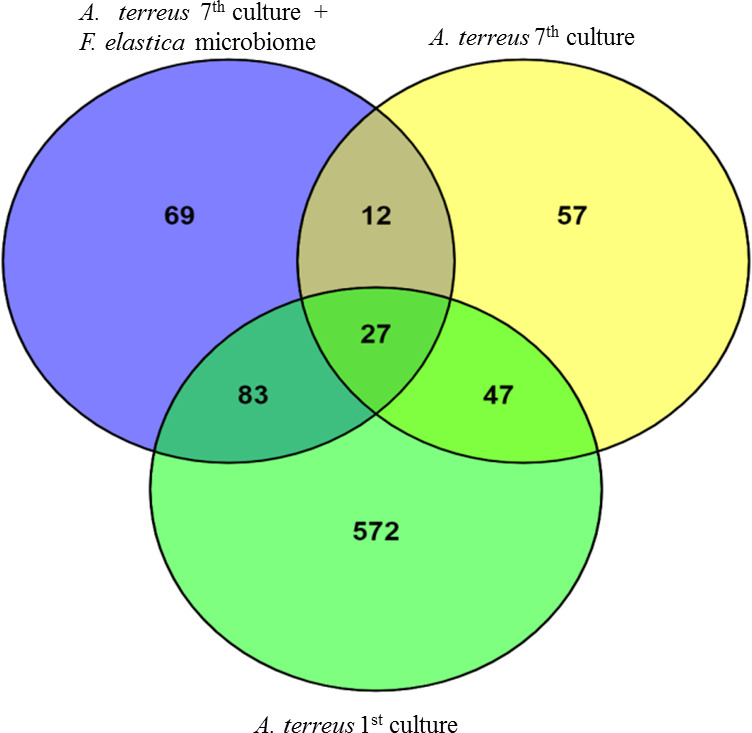
Venn diagrams of the differentially expressed proteins of the first and seventh cultural generations of *A. terreus* and seventh generation of *A. terreus* in response to 1.0% surface-sterilized leaves of *F. elastica*.

The differential expressed proteins were considered as highly significant if they exhibited a fold change ≥ 15 or ≤1 with *P* ≤ 0.05. The differentially abundant proteins were counted by 40 proteins, with approximately 27 upregulated proteins, as revealed from the Venn diagrams of the differentially abundant proteins of the first and seventh cultural generations and seventh generation of *A. terreus* in response to 1.0% leaves of *F. elastica* (Fig. 2).

**Fig 3:**
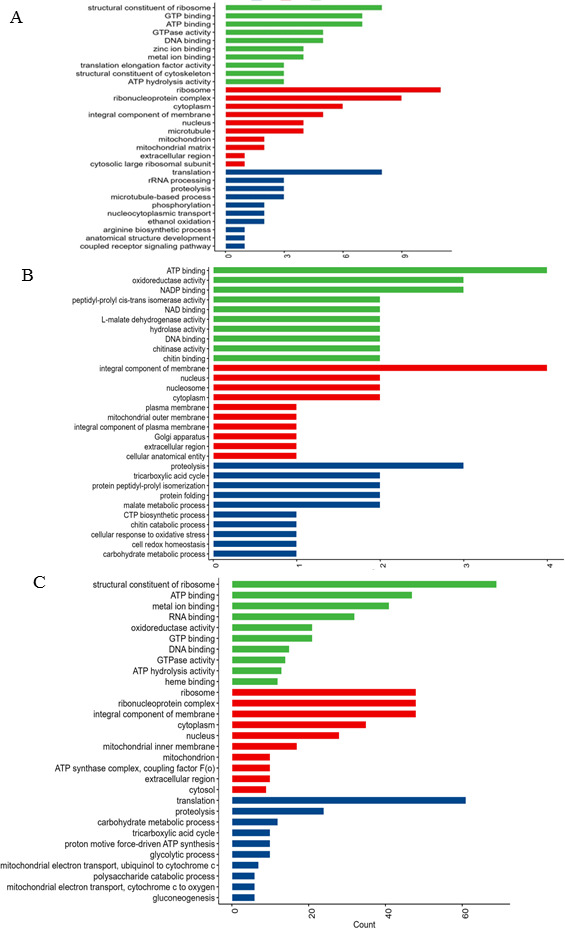
Gene Ontology analyses of the total proteome of the zero culture (**A**), seventh cultural generation (**B**), and seventh cultural generation amended with 1.0% leaves of *F. elastica* (**C**). The GO functional enrichment analysis showing the proteins of biological processes (PB), molecular functions (MF), and cellular compartments (CC).

**Fig 4:**
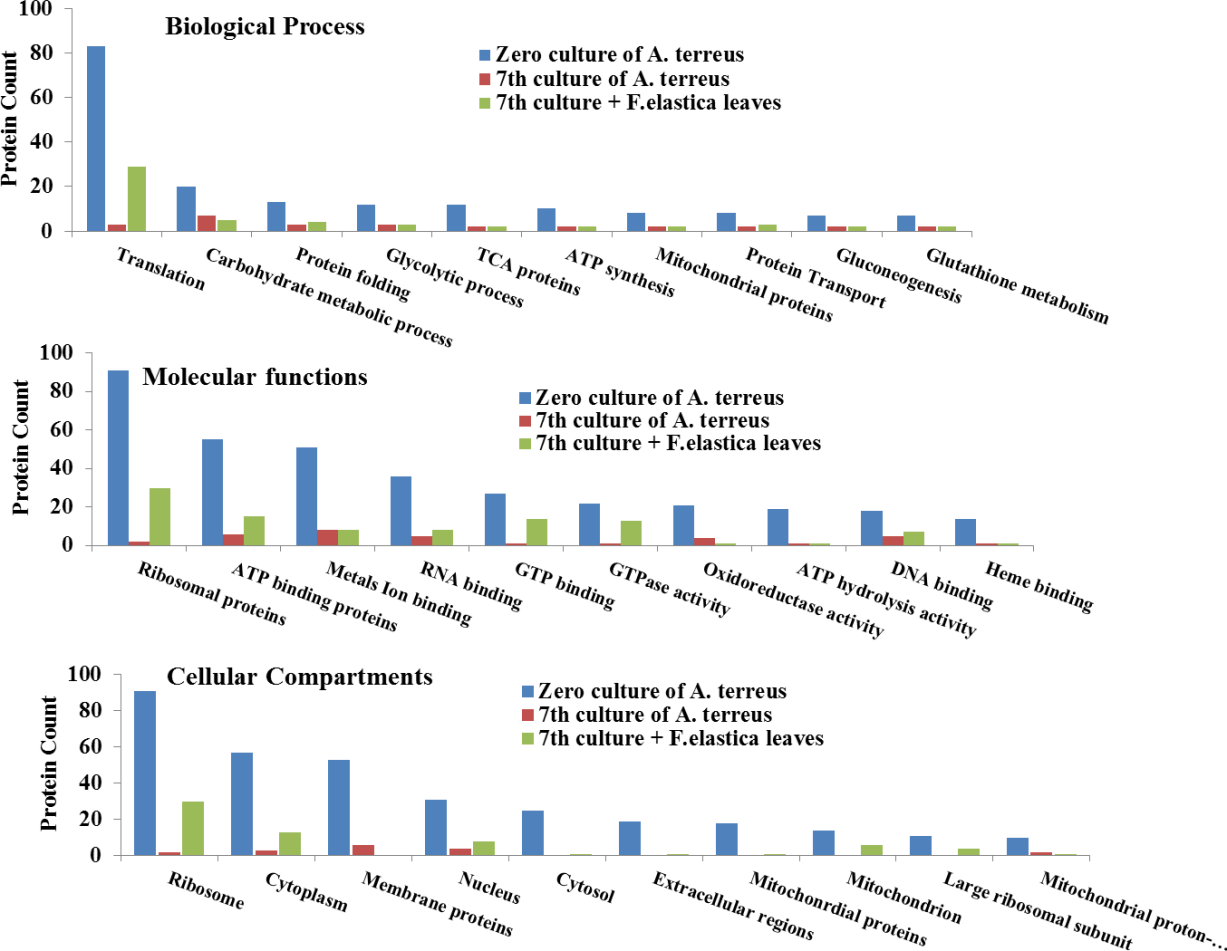
GO enrichment analyses showing the biological processes, molecular functions, and cellular compartments of the proteome of zero culture *A. terreus*, seventh generation, and seventh generation *A. terreus* amended with 1.0% leaves of *F. elastica*.

The GO enrichment analyses have been performed to categorize the differentially abundant proteins into biological processes, molecular functions, and cellular compartments of the zero culture, seventh subculture, and seventh subculture of *A. terreus* with 1% leaves of *F. elastica* (Fig. 3). The functional items of GO enrichment analysis in biological process were assessed for the translation, carbohydrate metabolism, protein folding, glycolytic process, TCA proteins, ATP synthesis, mitochondrial proteins, transport, gluconeogenesis, and glutathione metabolism of the zero culture, seventh generation, and seventh generation of *A. terreus* culture amended with 1% *F. elastica* microbiome. From the biological process, the highest functional GO terms of the seventh culture of *A. terreus* with leaves of *F. elastica* belong to translation, carbohydrate metabolic process, protein folding, and glycolytic process by about 29, 5, 4, and 3 differentially abundant proteins (Fig. 4). Also, from GO terms, the amounts of proteins involved with the biological processes, translation, carbohydrate metabolism, and protein folding of the zero culture of *A. terreus*, were 83, 20, and 13, respectively , with 3, 7, and 3 differentially abundant proteins on the seventh subculture of *A. terreus*, respectively. Thus, upon addition of *F. elastica* leaf parts, the biological process especially translation, carbohydrate metabolism, and protein folding potency was completely restored, in correlation with restoring the biosynthesis of CPT. In addition, from the GO analyses, the molecular functions process namely ribosomal proteins, ATP binding proteins, metal ion binding, RNA binding proteins, GTP binding proteins, and GTPase activity were the most affected proteins, with higher counts at the zero culture of *A. terreus*, with noticeable disappearance by the seventh culture of *A. terreus* and complete restoration with the addition of leaves *F. elastica* to the seventh culture of *A. terreus*. From the molecular function of GO terms, the ribosomal proteins and ATP binding proteins were the highly significant differentially abundant proteins in the zero culture of *A. terreus* (91 and 55 proteins), seventh culture generation of *A. terreus* (two and six proteins), and seventh culture amended with the leaves of *F. elastica* (30 and 15 proteins) (Fig. 4). Thus, it could be deduced that the attenuation and expression of the gene cluster encoding the secondary metabolites of fungi could be related to the ribosomal protein biogenesis. From the cellular compartments GO terms, the ribosome and cytoplasm biogenesis were the most differentially affected cellular compartments on the zero culture *A. terreus* (91 and 57 proteins), seventh culture of *A. terreus* (two and three proteins), and seventh culture amended with *F. elastica* leaves (30 and 13 proteins). So, ribosome biogenesis was the most affected cellular compartment with an obvious higher count on the zero culture of *A. terreus* and abolishing with the subculturing of *A. terreus* (two counts) and restoring upon addition of 1% *F*. *elastica* leaf parts (30 proteins) (Fig. 4). Thus, from the GO enrichment of the proteomic results, the proteins related to the translation process, ribosome functions and ribosome biogenesis were the most affected proteins as revealed from the biological process, molecular functions and cellular compartments enrichment analyses that mainly associated the metabolic attenuation and restoring of CPT biosynthesis by *A. terreus*.

### KEGG pathway analysis of the differentially abundant proteins and correlation with CPT biosynthesis by *A. terreus*

**Fig 5:**
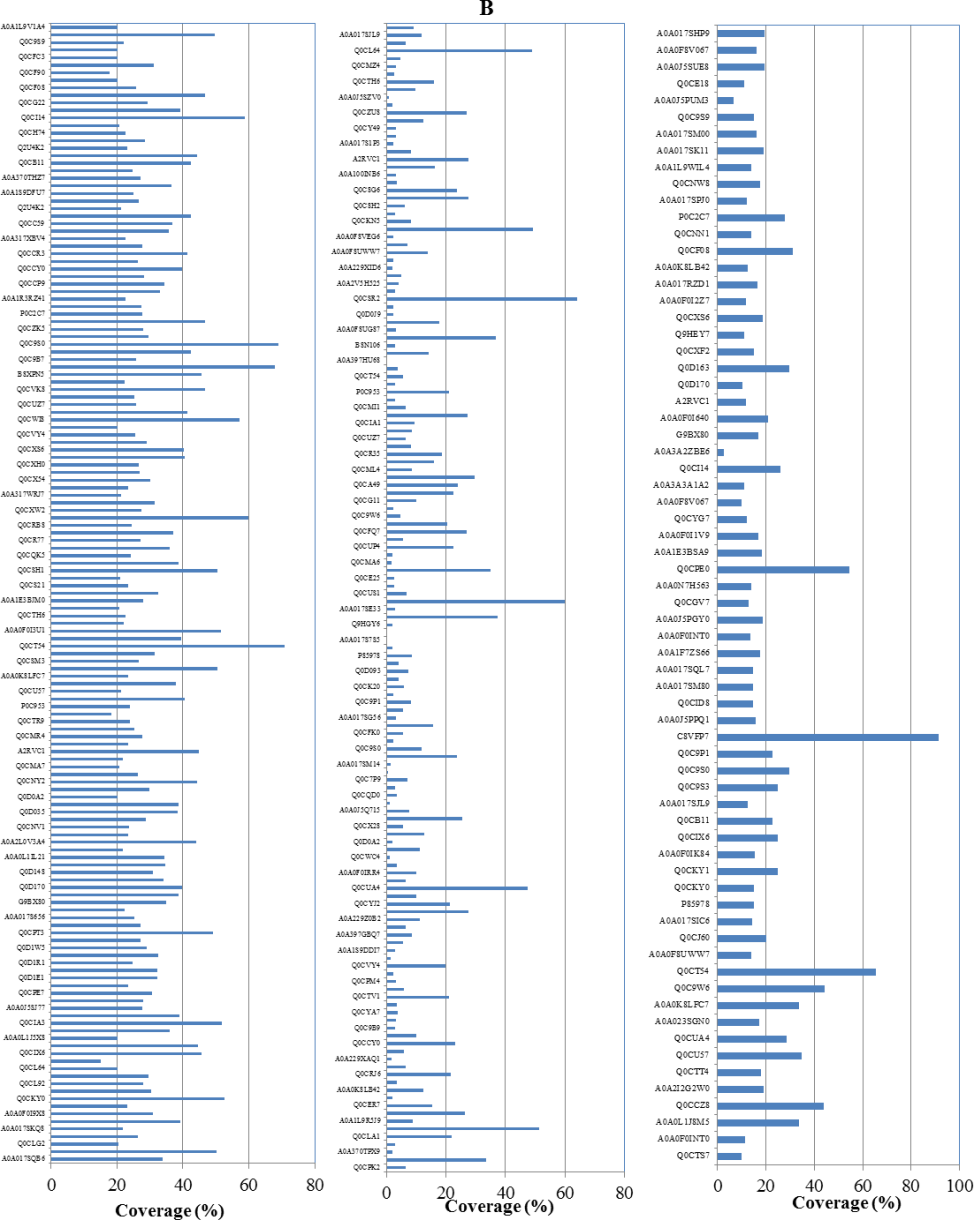
Gene Ontology and KEGG enrichment analysis of the differentially expressed proteins of the first culture of *A. terreus* (**A**), seventh culture of *A. terreus* (**B**), and seventh culture of *A. terreus* amended with microbiome of *F. elastica* (**C**).

**Fig 6:**
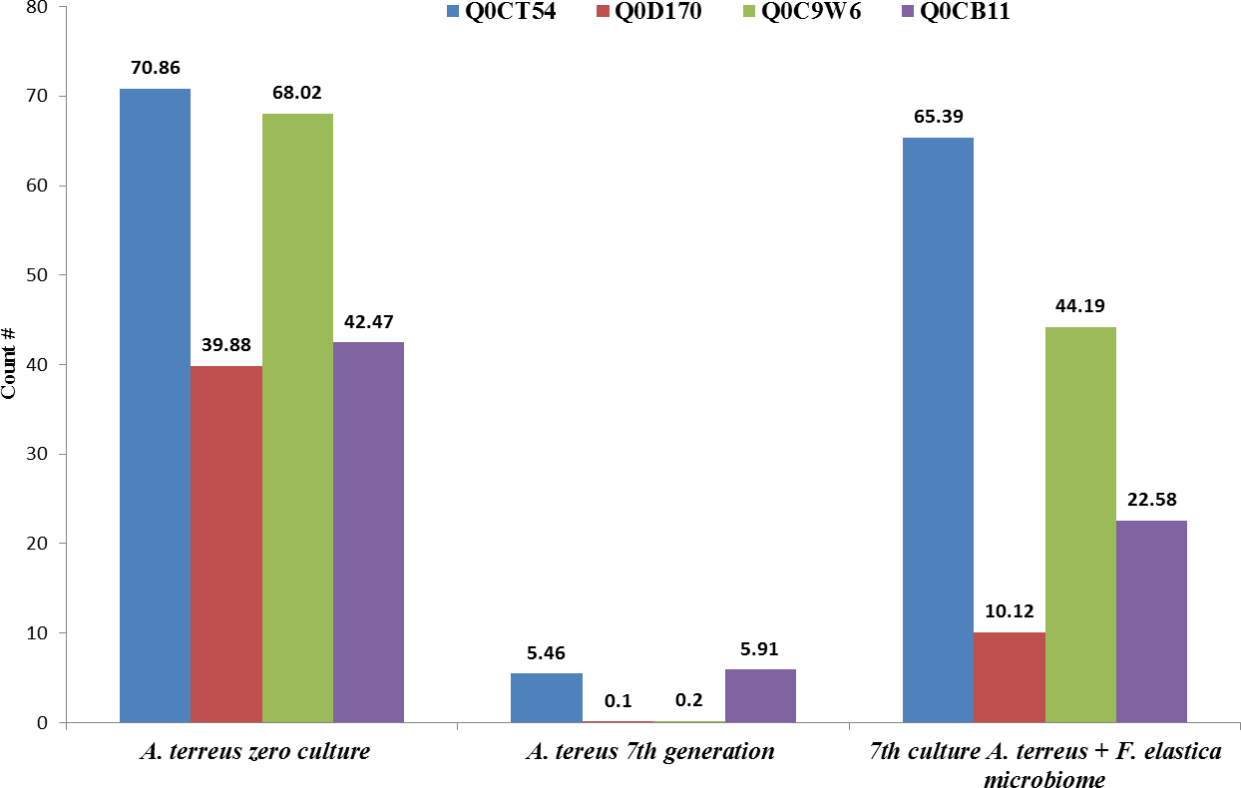
The most differentially expressed proteins from the zero culture *A. terreus* , seventh generation, and seventh generation of *A. terreus* amended with 1.0% leaves of *F. elastica*. The differentially expressed proteins were Q0CT54 hypothetical protein, Q0D170 60S ribosomal protein L6, Q0C9W6 peptidylprolyl cis-trans isomerase, and Q0CB11 predicted protein. The protein abundance was calculated based on counting and assignment to at least validated unique peptide and crossing signal to noise ratio (SNR) . The signal to noise ratio was above 20 and calculated by formula = 10 x log(signal/noise).

From the GO enrichment analyses, the ribosome biogenesis, ribosomal proteins, and protein of the translation processes could have a direct correlation with the synthetic metabolic machinery of CPT by *A. terreus*, as revealed from their attenuation and restoring upon addition of *F. elastica* microbiome. From the KEGG enrichment analysis, the differential proteins of the seventh generation of *A. terreus* with *F. elastica* microbiome were mainly associated with the molecular functions, especially secondary metabolite biosynthesis (Fig. 5). Also, the expression of several hypothetical proteins especially with IDs Q0CLA1, Q0CPT3, Q0CSM9, Q0CSH1, and Q0CY94, in addition to the protoplast secreted protein (Q0CWI8), was strongly attenuated with the successive subculturing of *A. terreus*, without any detectable restoration with the addition of *F. elastic* microbiome. These proteins mainly belong to the histidine triad (HIT) motif as being highly conserved in a variety of organisms. The attenuation of these proteins with the fungal subculturing without restoring to these proteins with the addition of *F. elastica* microbiome negates the direct implantation of these proteins with the secondary metabolite biosynthesis especially CPT. Additionally, the expression of several hypothetical proteins (Q0CKY0, Q0CI14, and C8VFP7), Woronin body protein (Q0CIA3), and nucleoside diphosphate kinase (Q0CE18) was initiated upon addition of microbiome of *F. elastica* leaves to the zero culture of *A. terreus*. In contrary to the overall decreasing of total proteome expression, the expression of hypothetical proteins (Q0CIJ2 and Q0CL64), conidiation-specific protein 10 (Q0CU25 and Q0CMC2), and predicted proteins (Q0CSR2) were dramatically increased by approximately 2-, 10-, and 60-fold, respectively, with the fungal subculturing (Fig. 5). The differentially abundant proteins that highly attenuated with the fungal subculturing were the hypothetical protein (Q0CT54), 60S ribosomal protein L6 (Q0D170), mitochondrial peptidylprolyl *cis*/*trans* isomerase (Q0C9W6), predicted proteins (Q0CB11), and DNA damage checkpoint protein rad24 (Q0C9S0) that were decreased by about 14-, 39-, 68-, 8, and 6-fold, respectively, with the seventh cultural generation of *A. terreus*. However, with the addition of the surface-sterilized leaves of *F. elastica* to the seventh generation of *A. terreus*, the molecular expression of the hypothetical protein, 60S ribosomal protein L6, mitochondrial peptidylprolyl *cis*/*trans* isomerase, predicted proteins, and DNA damage checkpoint protein rad24 was completely restored, in correlation with the restoration of CPT biosynthesis (Fig. 6). With the addition of *F. elastica* microbiome, the expression of 40S ribosomal protein, outer mitochondrial membrane porins, histone H2A, and peptidyl-prolyl *cis*/*trans* isomerase by *A. terreus* was significantly enriched. From the KEGG analysis of the differentially abundant proteins, hypothetical protein (Q0CT54), 60S ribosomal protein L6 (Q0D170), mitochondrial peptidylprolyl *cis*/*trans* isomerase (Q0C9W6), predicted proteins (Q0CB11), and DNA damage checkpoint protein rad24 (Q0C9S0), these proteins were related to terpenoid backbone biosynthesis (map00900), monoterpenoid biosynthesis (map00902), and biosynthesis of various alkaloids (map00996), respectively. From the GO enrichment analyses and Software Tool for Researching Annotations of Proteins (STRAP), the highest differentially expressed protein was annotated as Pleckstrin homology domain-containing protein (Q0CT54), mitochondrial peptidylprolyl *cis*/*trans* isomerase (Q0C9W6), 60S ribosomal protein L6 (Q0D170), and So-Cu domain-containing protein-predicted proteins (Q0CB11). From the combined UniProtK annotation and GO analysis, the PH domain-containing protein (Q0CT54) has a molecular function as a glycosyltransferase with higher distribution in various fungi. From the UniProt annotation analysis, the Sod-Cu domain-containing protein (Q0CB11) has a molecular function of metal binding and biological process of superoxide metabolic process with copper as ligand. Also, mitochondrial peptidylprolyl *cis*/*trans* isomerase (Q0C9W6) is one of the most differentially abundant proteins that was attenuated with the fungal subculturing and restored by the microbiome of *F. elastica*; this protein has a crucial role in protein folding by catalyzing the *cis*/*trans* isomerization of proline imidic peptide bonds in proteins. Conclusively, from the GO enrichment, KEGG enrichment, and UniProt annotation analyses, the most differentially expressed proteins that significantly attenuated with the fungal subculturing are the PH-domain-containing protein, mitochondrial peptidylprolyl *cis*/*trans* isomerase, 60S ribosomal protein L6 and Sod-Cu domain-containing protein.

### Protein-protein interaction analyses

**Fig 7:**
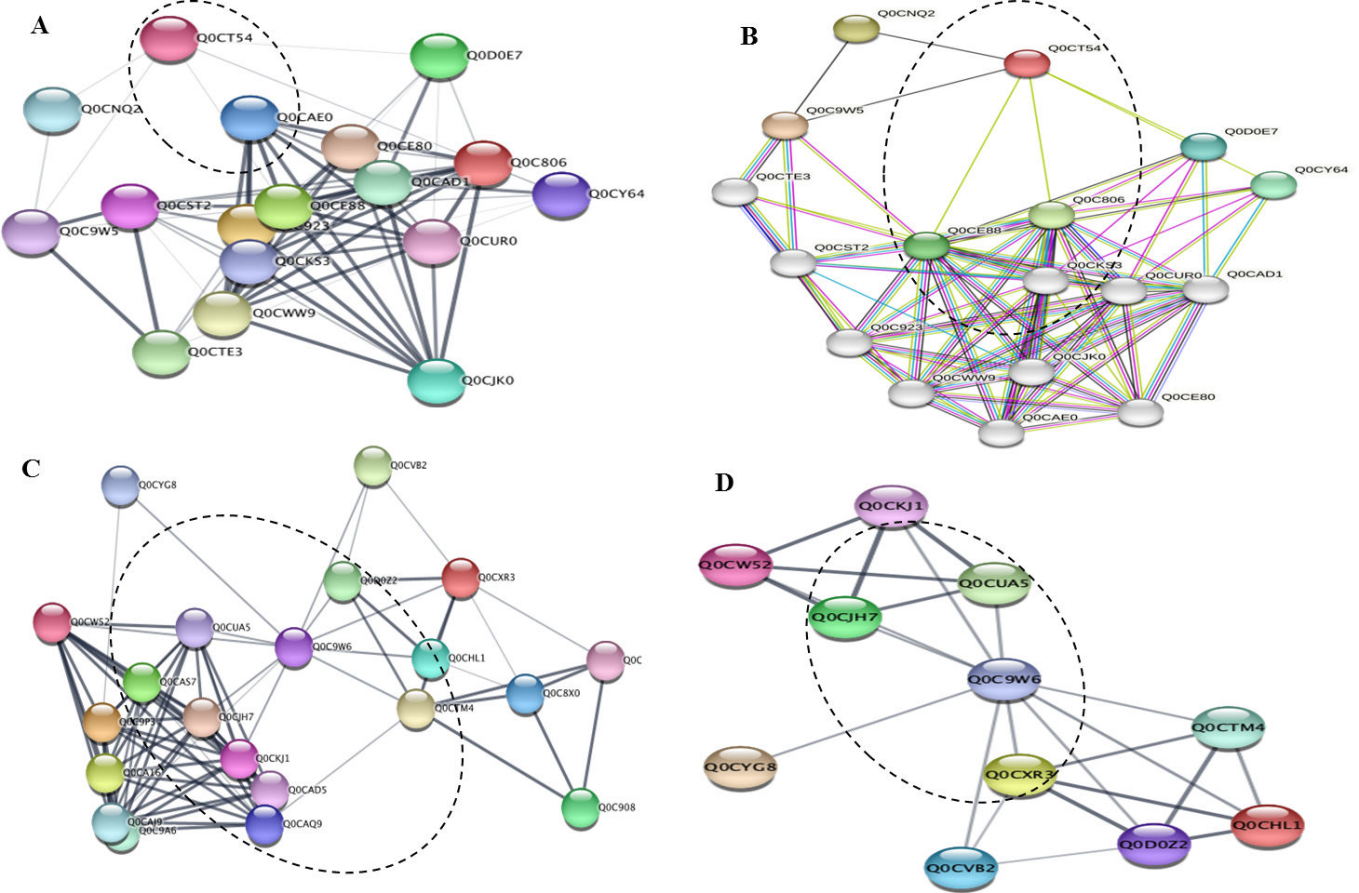
Protein-protein interactions predicted for DEPs found in *A. terreus* in response to amendment with microbiome of *F. elastica*. The STRING database was used to examine the differentially expressed proteins Q0CT54 (**A and B**) and Q0C9W6 (**C and D**) of *A. terreus* in response to addition of *F. elastica* microbiome. Each node in the network represents a distinct DEP. Interactions are shown by the colored lines connecting each node with the weight of each line representing the confidence of the interaction based on available evidence in the database. Clusters of interest are indicated by the colored labels.

**Fig 8:**
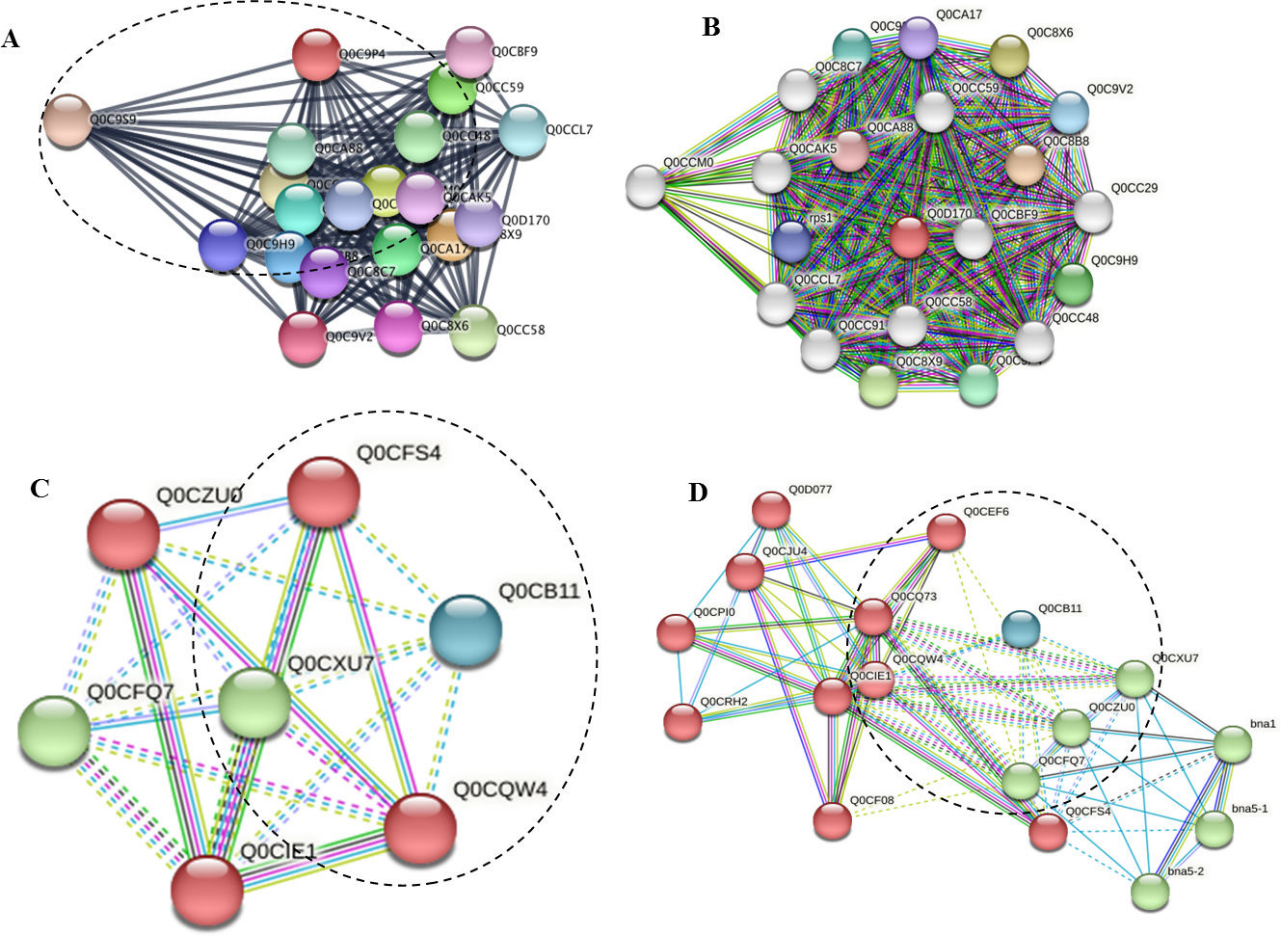
Protein-protein interactions predicted for differentially expressed proteins in *A. terreus* in response to microbiome of *F. elastica*. The STRING database was used to examine the differentially expressed proteins Q0D170 (**A and B**) and Q0CB11 (**C and D**) of *A. terreus* in response to *F. elastica* microbiome. Each node in the network represents a distinct DEP; the interactions are shown by the colored lines connecting each node with the weight of each line representing the confidence of the interaction based on available evidence in the database. Clusters of interest are indicated by the colored labels.

To explore the interaction metabolic pathways of the differentially abundant proteins of *A. terreus* with the subculturing and amendment with *F. elastica* microbiome, the target proteins were annotated by STRING “functional protein association networks” software package ver.11.5 (https://string-db.org/). For the PH domain-containing protein of *A. terreus*, the branches of the interaction network were related to the molecular functions and biological compartments of histone H4 (Q0D0E7) as a core component of nucleosome wrapping the DNA into chromatin, regulating the DNA accessibility to the subsequent cellular expression machineries. Histone H4 plays a central role in transcriptional regulation, DNA repair, DNA replication, and chromosomal stability. Also, the PH domain-containing protein of *A. terreus* has a direct correlation with the heat shock proteins 70 kDa (Q0C806) and 82 kDa (Q0CE88) that act as a chaperone for the proper protein folding (Fig. 7). Also, from the protein-protein interaction analysis, the mitochondrial peptidylprolyl *cis*/*trans* isomerase (Q0C9W6) has a direct relation to the GTPase-activating protein 1, Ubiquitin-conjugating enzyme E2-18 kDa, Cytochrome c, mitochondrial membrane porins, and heat shock proteins (Fig. 7). From the protein-protein interaction analysis, the 60S ribosomal protein L6 (Q0D170) mainly interacts with the 60S ribosomal protein L4-2 (ATEG_09279), 60S ribosomal protein L7 (ATEG_02206), 60S ribosomal protein L18 (ATEG_06847), 60S ribosomal protein L32-A (ATEG_02305), 40S ribosomal protein S14 (ATEG_01528), 40S ribosomal protein S14 (ATEG_01528), and 60S ribosomal protein L14-A (ATEG_07079) (Fig. 8). In addition, from the protein-protein interaction analysis, the Sod-Cu domain-containing protein (Q0CB11) has a direct molecular and metabolic correlation with the peroxisomal catalase (Q0CFS4), superoxide dismutase (Q0CQW4), and catalase (Q0CFQ7). This system of antioxidant destroys the toxic free radicals that are normally produced within the cells. From the functional enrichment analysis of STRING software package, based on the false discovery rate, the Sod-Cu domain-containing protein has a direct implication on the reactive oxygen species metabolism, cellular detoxification, response to oxidative stress, and superoxide dismutase activity. From the KEGG pathways analysis, the Sod-Cu domain-containing protein has a direct relation to the peroxisome (map04146), tryptophan metabolism (map00380), MAPK signaling pathway (map04011), and glyoxylate and dicarboxylate metabolism (map00630). Also, from the UniProt annotation, the Sod-Cu domain-containing protein has a direct correlation with the peroxidase, oxidoreductase, hydrogen peroxide, and pyridine nucleotide biosynthesis.

### Metabolomics profile analyses of *A. terreus* in response to amendment with *F. elastica* microbiome

**Fig 9:**
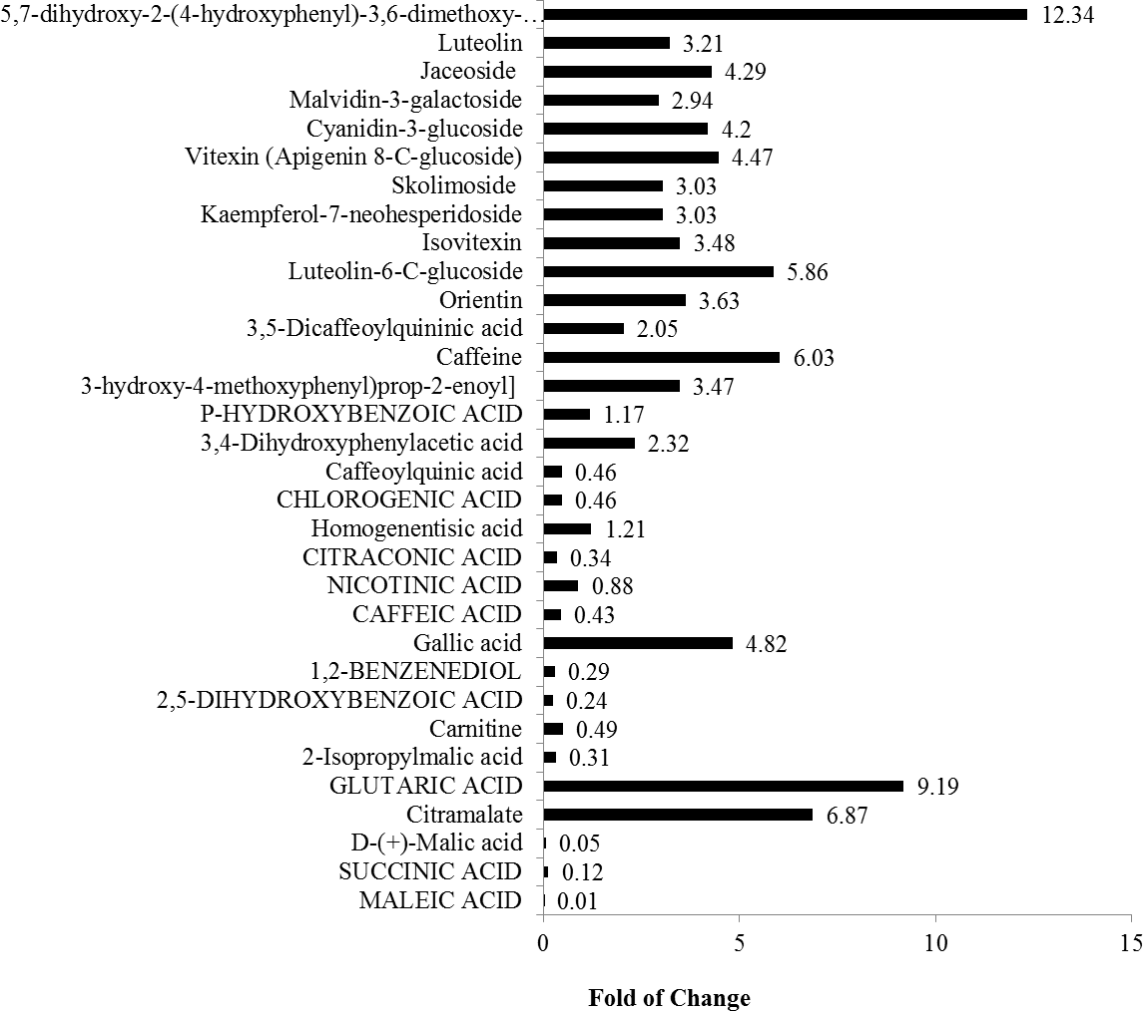
Metabolic profiling of the seventh culture of *A. terreus* in response to microbiome of *F. elastica*. The differentially expressed metabolites.

The total ion chromatogram (TIC) and base peak chromatogram (BPC) of LC-MS/MS acquisitions of *A. terreus* metabolites in response to amendment with *F. elastica* microbiome were shown. From the TIC and BPC, a significant difference in the metabolic profile of the seventh cultural generation of *A. terreus* and in response to amendment with the microbiome of *F. elastica* was shown. The TIC and BPC of the two samples were overlapped; the red- and blue-colored chromatograms referred to the seventh culture of *A. terreus* and seventh culture amended with *F. elastica* microbiome (Fig. 9). Among the annotated metabolites, 50 metabolites in *A. terreus* were reported to be differentially fluctuated in response to the amendment with *F. elastica* microbiome, as revealed from the ESI + and ESI− modes. Practically, the differentially abundant metabolites mainly belong to flavonoids, dicarboxylic acid derivatives, and hydroxyl fatty acids. The metabolites, molecular formula, MS/MS fragmentation, and metabolic functions were listed in Table 1. The expression of the metabolites 5,7-dihydroxy-2-(4-hydroxyphenyl)-3,6-dimethoxy-4H-chromen, glutaric acid, citramalate, and caffeine by *A. terreus* was increased by about 12.34-, 9.19-, 6.87-, and 6.03-fold upon amendment with *F. elastica* microbiome. Also, the expression of several metabolites mainly cyanidin-3-glucoside, vitexin (apigenin 8-C-glucoside), luteolin-6-C-glucosiden, gallic acid, kaempferol-7-neohesperidoside, isovitexin, and orientin by *A. terreus* was mainly increased by about three- to fivefold upon addition of *F. elastica* microbiome (Fig. 9). The most significantly abundant metabolites by *A. terreus* in response to *F. elastica* microbiome were mainly belonging to flavonoid C-glycosides, flavonoid 7-O-glycosides, and flavonoid 8C glycosides. From the KEGG database, the differential metabolites were linked to the specific metabolic pathways to explore the significant metabolic pathways using enrichment analysis and study the dynamic changes of secondary metabolite production by *A. terreus* in response to amendment with *F. elastica* microbiome. Most of the differentially abundant metabolites were mainly involved in the glycolysis, TCA cycle, mevalonate pathway, terpenoid synthesis, and shikimate pathways, as revealed from the metabolic interactions derived from the KEGG pathway mapper (Fig. 10). Several metabolites from *A. terreus* were significantly induced upon addition of *F. elastica* microbiome with direct correlation to the aromatic amino acid, alkaloid, and terpenoid synthetic pathways in addition to the camptothecin biosynthetic pathway. Upon addition of *F. elastica* microbiome, several intermediates were induced mainly acetyl-CoA, α-ketoglutarate, acetoacetyl-CoA, isoperene units, subsequently geranyl-pyrophosphate, geraniol, and ultimately camptothecin secondary metabolite. Also, the synthesis of aromatic amino acids by *A. terreus* was significantly increased upon addition of *F. elastica* microbiome that might have a direct interaction with the synthesis of tryptophan and subsequently with the camptothecin biosynthesis. Practically, the over-induced metabolites by *A. terreus* in response to *F. elastica* microbiome have multiple interactions with the glycolysis, TCA cycles, terpenoid and alkaloid biosynthesis, and various metabolites.

**TABLE 1:** Metabolic profiling of *A. terreus* by LC-MS/MS in response to amendment with *F. elastica* microbiome

#	Rt (min)	Precursor (m/z)	Formula	Error (ppm)	Adduct ion name	MS/MS	Metabolite name	Ontology	Sample (1:2)
1	1.09	115.0037	C_4_H_4_O_4_	0.4	[M-H]-	71.0136, 87.0082	MALEIC ACID	Dicarboxylic acid derivative	0.01
2	1.1	117.0193	C_4_H_6_O_4_	0.1	[M-H]-	73.0299,99.0094	SUCCINIC ACID	Dicarboxylic acid derivatives	0.12
3	1.1	133.0143	C_4_H_6_O_5_	0.8	[M-H]-	71.0138, 72.9931, 89.0260, 115.0440	D-(+)-Malic acid	Beta hydroxy acid derivatives	0.05
4	1.1	147.0299	C_5_H_8_O_5_	0.8	[M-H]-	85.0290, 87.0086,	Citramalate	Hydroxy fatty acid	6.87
5	1.12	131.035	C_5_H_8_O_4_	0	[M-H]-	69.0343, 87.0446, 113.0242	GLUTARIC ACID	Dicarboxylic acid derivatives	9.19
6	1.19	175.0612	C_7_H_12_O_5_	0.1	[M-H]-	85.0660, 113.0612 115.0403	2-Isopropylmalic acid	Hydroxy fatty acid	0.31
7	1.21	162.1125	C_7_H_15_NO_3_	0.2	[M + H]+	58.0640, 60.0794, 85.0269, 102.0893,	Carnitine	Carnitines	0.49
8	1.23	153.0193	C_7_H_6_O_4_	0.7	[M-H]-	91.0206, 108.0231, 109.0307	2,5-DIHYDROXYBENZOIC ACID	Hydroxybenzoic acid derivatives	0.24
9	1.26	109.0295	C_6_H_6_O_2_	0.4	[M-H]-	81.0321, 91.0170, 108.0198,	1,2-BENZENEDIOL	Catechols	0.29
10	1.32	169.0143	C_7_H_6_O_5_	0.1	[M-H]-	69.0362, 79.0199, 95.0132, 107.0155,	Gallic acid	Gallic acids	4.82
11	1.34	179.035	C_9_H_8_O_4_	0.1	[M-H]-	89.0447, 117.0389, 134.0387, 135.0449	CAFFEIC ACID	Hydroxycinnamic acids	0.43
12	1.36	122.0248	C_6_H_5_NO_2_	6.6	[M-H]-	78.0347, 94.0251	NICOTINIC ACID	Pyridine carboxylic acids	0.88
13	1.36	129.0193	C_5_H_6_O_4_	0.6	[M-H]-	57.0335, 85.0284	CITRACONIC ACID	Methyl-branched fatty acids	0.34
14	1.36	167.035	C_8_H_8_O_4_	0.4	[M-H]-	108.0217, 122.0371	Homogentisic acid	2(hydroxyphenyl)acetic acids	1.21
15	1.36	353.0878	C_16_H_18_O_9_	0.3	[M-H]-	135.044, 161.0233, 179.0346,	CHLOROGENIC ACID	Quinic acids and derivatives	0.46
16	1.36	353.0878	C_16_H_18_O_9_	0.3	[M-H]-	135.0457, 179.0353, 191.0565	Caffeoylquinic acid	Quinic acids and derivatives	0.46
17	2.5	167.035	C_8_H_8_O_4_	0.9	[M-H]-	93.0347, 121.0298, 123.0454, 137.0242	3,4-Dihydroxy-phenylacetic acid	Catechols	2.32
18	2.62	137.0244	C_7_H_6_O_3_	0.3	[M-H]-	65.0398, 93.0345	P-HYDROXYBENZOIC ACID	Hydroxybenzoic acid derivatives	1.17
19	3.72	367.1035	C_17_H_20_O_9_	0.1	[M-H]-	134.0372, 173.0453, 193.0513	1,3,5-trihydroxy −4- [(E)−3-(methoxyphenyl)	NA	3.47
20	4.65	195.0877	C_8_H_10_N_4_O_2_	0.5	[M + H]+	69.0424, 83.0612, 110.0709	Caffeine	Xanthines	6.03
21	5.39	515.1195	C_25_H_24_O_12_	0.6	[M-H]-	135.049, 179.0346, 191.0547, 294.9011	3,5-Dicaffeoylquininic acid	Quinic acids and derivatives	2.05
22	5.45	447.0933	C_21_H_20_O_11_	0.4	[M-H]-	294.093, 297.0378, 327.0501	Orientin	Flavonoid 8-C-glycosides	3.63
23	5.98	449.1078	C_21_H_20_O_11_	0.6	[M + H]+	287.054, 299.0553, 329.0664, 353.0636	Luteolin-6-C-glucoside	Flavonoid C-glycosides	5.86
24	6.06	431.0984	C_21_H_20_O_10_	0.4	[M-H]-	283.0607,311.0546, 341.0663,	Isovitexin	Flavonoid C-glycosides	3.48
25	6.55	593.1512	C_27_H_30_O_15_	0.1	[M-H]-	285.0387	Kaempferol-7-neohesperidoside	Flavonoid-7-O-glycosides	3.03
26	6.55	593.1512	C_27_H_30_O_15_	0.1	[M-H]-	285.0387	Skolimoside	Flavonoid-7-O-glycosides	3.03
27	6.57	433.1129	C_21_H_20_O_10_	0	[M + H]+	283.060, 313.0719, 337.0735, 415.1011	Vitexin (Apigenin 8-C-glucoside)	Flavonoid 8-C-glycosides	4.47
28	6.79	449.104	C_21_H_21_O_11_	0.5	[M]+	287.0548	Cyanidin-3-glucoside	Anthocyanidin-3-O-glycosides	4.20
29	7.76	492.1273	C_23_H_25_O_12_	0.6	[M-2H]-	271.025, 285.0362, 299.0197, 313.0325, 329.0632, 461.0652	Malvidin-3-galactoside	Anthocyanidin-3-O-glycosides	2.94
30	7.78	491.1195	C_23_H_24_O_12_	0.6	[M-H]-	218.9489, 271.024, 313.0348, 328.0563, 445.1882, 476.0931	Jaceoside	Flavonoid-7-O-glycosides	4.29
31	9.2	285.0405	C_15_H_10_O_6_	0.2	[M-H]-	107.011, 132.0208, 133.0304, 151.0043, 175.0368	Luteolin	Flavones	3.21
32	10.35	329.0667	C_17_H_14_O_7_	0.5	[M-H]-	271.0245, 299.0192, 314.0429	5,7-dihydroxy-2-(4-hydroxyphenyl)-3,6-dimethoxy-4H-chromen	6-O-methylated flavonoids	12.34

**Fig 10:**
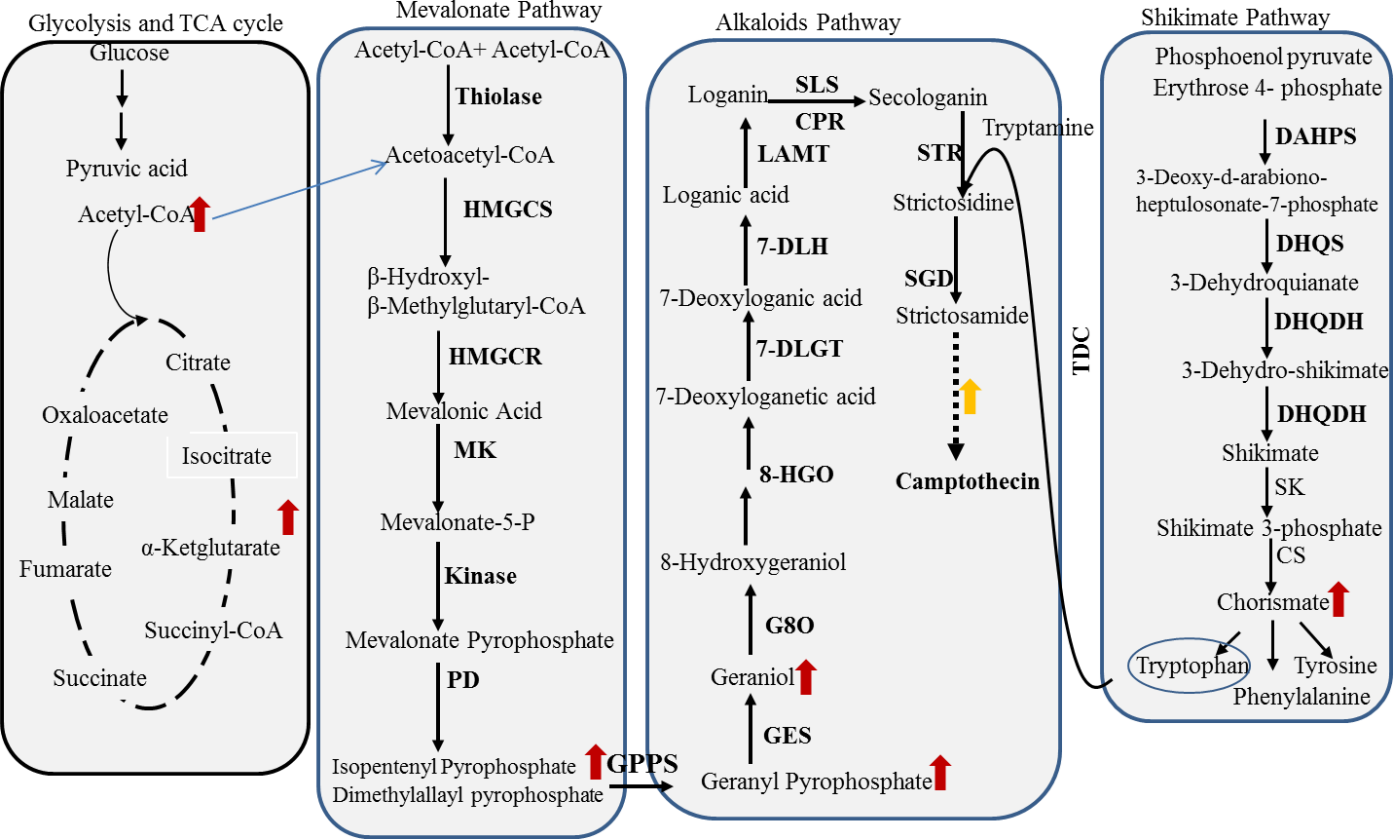
Metabolic network between the specific metabolites and KEGG pathways. The red arrows indicate the increase in the content of these intermediate compounds by *A. terreus* in response to microbiome of *F. elastica*.

## DISCUSSION

Camptothecin and its chemical derivatives are one of the most commercial anticancer drugs with broad-range activity toward various tumor cells. Regarding the difficulties to fulfill the needed amounts of CPT from the natural sources, exploring the potency of endophytic fungi for CPT production raise the hope for commercial CPT production, due to their fast growth rates, feasibility of bulk biomass, and metabolic manipulation (15, 17, 18, 31, 35, 49). However, attenuation of the biosynthetic machinery of CPT by fungi with subculturing and storage is the main challenge that impedes their usage to be an industrial platform (10, 21, 50). This loss of CPT productivity with subculturing of fungi could be due to the reprogramming of fungal cellular physiology and silencing of rate-limiting genes or transcriptional factors of CPT biosynthesis (10, 51, 52). Practically, the biosynthetic machinery of CPT by endophytic fungi has been completely restored upon addition of the entire microbiome of the host plants, ensuring the secretion of a specific chemical signal from the CPT-producing and/or non-CPT-producing endogenous microbes of the host plant that triggers the CPT biosynthetic gene cluster of the target fungi (9, 16, 53, 54). However, the mechanism of attenuation of the expression of the CPT-encoding gene cluster upon fungal subculturing, in addition to the restoration of the CPT biosynthetic machinery upon addition of the plant microbiome, remains ambiguous. So, the objective of this work was to explore the molecular machinery of CPT biosynthesis in response to fungal subculturing and addition of *F. elastica* microbiome based on the proteomics and metabolic analyses.

The yield of CPT by *A. terreus* was sequentially reduced with the fungal subculturing, by the seventh culture; the yield of CPT by *A. terreus* was reduced by >50%, compared with the zero culture. A strong correlation was observed for the expression of the CPT biosynthetic rate-limiting genes “*sls* gene” and the overall yield of CPT. Secologanin synthase is one of the committed enzymes of CPT biosynthesis, catalyzing the oxidative cleavage of the cyclopentane ring in loganin to form secologanin, in addition to geraniol synthase, strictosidine synthase, strictosidine β-glucosidase, and tryptophan decarboxylase (17, 55). Coincidently, the CPT biosynthetic attenuation with the fungal subculturing under axenic monoculture conditions and storage was reported (22, 33, 56, 57). This weakening of CPT yield was hypothesized to the horizontal transfer of the CPT biosynthetic genes from the host plant to their endophytic fungi with random mutations, and the attenuated expression of the fungal CPT biosynthesis with the subculturing could be due to the dilution of the specific elicitors from the host plant (10, 22, 33, 51, 56, 57). Nevertheless, this hypothesis was negated since the biosynthetic gene clusters of CPT are physically located on the genome of the producing fungi; nevertheless, their expression is dependent on the chemical signals derived from the plants or their endogenous microbiome, that is, the most reasonable hypothesis. Thus, the attenuation of CPT biosynthetic machinery could be due to the dilution of the plant-derived signals with successive culturing and/or even autonomous degradation of the spore-associated signals with the fungal storage.

In an endeavor to assess the restoring potency of the CPT-encoding gene cluster of *A. terreus*, the seventh culture of *A. terreus* was amended with the microbiome of *F. elastica* leaves. Interestingly, the productivity of CPT by the seventh generation of *A. terreus* was exponentially improved with addition of the surface-sterilized *F. elastica* leaves by ~twofold compared with *A. terreus* zero culture. This noticeable restoration of the attenuated CPT biosynthetic machinery of *A. terreus* might be due to the release of CPT-producing/or non-CPT-producing endophyte from the plant tissue, with subsequent intimate co-growth with *A. terreus*, restoring the biosynthetic machinery of *A. terreus* CPT (22, 56, 57). The chemical compounds extracted from *F. elastica* with different solvents had no inducing response on the CPT yield, authenticating the dependence of the CPT biosynthetic machinery of *A. terreus* on encrypted biological signals derived from the microbiome of the host plant (9, 15, 22, 56, 57). A noticeable induction to the expression of sls was obsrved by about five fold compared to seventh generation of *A. terreus* that being matched with the metabolic analysis of CPT by HPLC. Thus, the attenuation of the molecular machinery of CPT biosynthesis could be related to the upstream enzymatic machinery of SLS enzyme, but not practically related to the SLS downstream process. Conclusively, the re-expression of the CPT biosynthetic genes could be due to the cultural communication and cross-talking among the CPT-producing and non-producing microbial endophytes. Similar metabolic observations were reported for taxol biosynthesis by *A. terreus* upon incorporation with microbiome of *Podocarpus gracilior (P. gracilior)* (27, 35). Consistently, the taxol yield by *Aspergillus flavipes (A. flavipes)* has been dramatically increased upon co-culturing with *Bacillus subtilis* that had an obvious antifungal activity (14, 55). Thus, the presence of specific endophytes and their intimate/physical interaction with *A. terreus* could be the pivotal signal for expression of the CPT-encoding genes.

To explore the differentially abundant proteins associated with the subculturing of *A. terreus* in correlation with attenuation and restoration of the productivity of CPT, the total proteome of the zero culture, seventh generation, and seventh generation with the microbiome of *F. elastica* was analyzed. The total intracellular proteins from the fungal cultures were extracted for the proteomic analysis. The significant differentially abundant proteins were counted by 40 proteins. From the GO enrichment analyses, the functional items of the biological process including the translation, carbohydrate metabolism, protein folding, glycolytic process, TCA proteins, ATP synthesis, mitochondrial proteins, gluconeogenesis, and glutathione metabolism significantly fluctuated among the fungal subculturing and amendment with *F. elastica* leaves. From the GO analyses of the seventh culture of *A. terreus* with the plant leaves, the functional terms that belong to the translation, carbohydrate metabolic process, protein folding, and glycolytic process were significantly upregulated. Upon addition of *F. elastica* leaves, biological processes especially translation, carbohydrate metabolism, and protein folding potency have been completely restored, correlating with restoration of the biosynthetic machinery of CPT. From the GO analyses, the molecular functions process ribosomal proteins, ATP binding proteins, metal ion binding, RNA binding proteins, GTP binding proteins, and GTPase activity were the significantly affected proteins, with high intensity at the first *A. terreus* culture with a noticeable abolishing by the seventh *A. terreus,* and complete restoring with the addition of the leaves of *F. elastica*. So, the attenuation of the gene cluster encoding the fungal secondary metabolites could be related to the ribosomal protein biogenesis, as observed from the dramatic fluctuation from the zero culture of *A. terreus*, abolishing with the subculturing and restoring upon addition of microbiome of *F. elastica*. From the KEGG enrichment, the differentially abundant proteins that highly downregulated with the fungal subculturing were hypothetical protein Q0CT54, 60S ribosomal protein L6 Q0D170, mitochondrial peptidylprolyl *cis*/*trans* isomerase Q0C9W6, predicted proteins Q0CB11, and DNA damage checkpoint protein rad24 Q0C9S0 that significantly decreased with the seventh culture of *A. terreus*. However, by addition of the microbiome of *F. elastica*, the molecular expression of these proteins was completely restored, in correlation with restoring CPT biosynthesis. From the KEGG analysis, the proteins Q0CT54, 60S ribosomal protein L6 Q0D170, mitochondrial peptidylprolyl *cis*/*trans* isomerase Q0C9W6, proteins Q0CB11, and DNA damage checkpoint protein rad24 Q0C9S0, were associated with terpenoids backbone biosynthesis map00900 and map00902, and alkaloids synthesis map00996. From the STRING and UniProt annotations, the highest differentially abundant protein Q0CT54 was annotated as Pleckstrin homology domain-containing protein, followed by the mitochondrial peptidylprolyl *cis*/*trans* isomerase Q0C9W6, 60S ribosomal protein L6 Q0D170, and So-Cu domain-containing proteins (Q0CB11). The molecular function of the PH domain-containing protein is a glycosyltransferase distributed in almost all fungi (58, 59). From the UniProt analysis, the Sod-Cu domain-containing protein has a molecular function of metal binding and biological process of superoxide metabolic process (60). The peptidylprolyl *cis*/*trans* isomerase is one of the most differentially abundant proteins, attenuated with the fungal subculturing, and completely restored with the microbiome of *F. elastica*, and this proteins has a crucial role in protein folding by catalyzing the *cis*/*trans* isomerization of proline imidic peptide bonds. From the GO enrichment, KEGG enrichment, and UniProt annotation, the most significant differentially proteins were the PH domain-containing protein, mitochondrial peptidylprolyl *cis*/*trans* isomerase, 60S ribosomal protein L6, and Sod-Cu domain-containing protein (60, 61). For the PH domain-containing protein, the interaction networks were related to the molecular functions and biological compartments of histone H4 (Q0D0E7) as a core component of nucleosome wrapping the DNA into chromatin, regulating the DNA accessibility to the subsequent cellular expression machineries (61). The PH domain-containing protein of *A. terreus* has a direct correlation with the heat shock proteins 70 kDa (Q0C806) and 82 kDa (Q0CE88) that act as a chaperone for the proper protein folding. From the protein-protein interaction analysis, the mitochondrial peptidylprolyl *cis*/*trans* isomerase (Q0C9W6) has a direct relation to the GTPase-activating protein, ubiquitin-conjugating enzyme E2-18, cytochrome c, mitochondrial membrane porins, and heat shock proteins. The 60S ribosomal protein L6 (Q0D170) has been noticed to be interacted with the ribosomal proteins L4-2 (ATEG_09279), protein L7 (ATEG_02206), protein L18 (ATEG_06847), protein L32-A (ATEG_02305), protein L14-A (ATEG_07079), 40S ribosomal protein S14 (ATEG_01528). Also, from the protein-protein interaction, the Sod-Cu domain-containing protein has a direct molecular and metabolic correlation with peroxisomal catalase, superoxide dismutase, catalase, peroxisome, tryptophan metabolism, MAPK signaling pathway, glyoxylate, and dicarboxylate metabolism. Also, from the UniProt annotation, the Sod-Cu domain-containing protein has a direct correlation with the peroxidase, oxidoreductase, hydrogen peroxide, and pyridine nucleotide biosynthesis. Consistently, the concentrations of secondary metabolites, antioxidant enzyme expression, and carbohydrate metabolism by *A. flavus* were significantly increased in response to the oxidative stress (62, 63). Similarly, the higher level of stress by H_2_O_2_ and the higher aflatoxin production by *A. flavus* NRRL3357 exhibited a much greater number of DEPs, ensuring the correlation between aflatoxin production levels and oxidative stress tolerance. From the GO enrichment and proteomic and metabolic interaction analyses, more extensive interaction networks for CPT biosynthesis was resolved by *A. terreus* in response to addition of *F. elastica* microbiome. Triggering of the rate-limiting enzymes of CPT biosynthesis by *A. terreus* could be due to the microbial interactions with endogenous microorganisms, *via* releasing of specific signals eliciting the biosynthetic machinery of CPT as verified from the chromatographic, proteomic, and metabolic analyses (15, 17, 36, 55).

From the metabolic analysis, the metabolites 5,7-dihydroxy-2-(4-hydroxyphenyl)-3,6-dimethoxy-4H-chromen, glutaric acid, citramalate, and caffeine by *A. terreus* were significantly increased by 12.34-, 9.19-, 6.87-, and 6.03-fold upon addition of *F. elastica* microbiome. Also, the expression of cyanidin-3-glucoside, vitexin, luteolin-6-C-glucosiden, gallic acid, kaempferol, isovitexin, and orientin by *A. terreus* was increased by about three- to fivefold upon addition of *F. elastica* microbiome. Most of differentially abundant metabolites were mainly involved in the glycolysis, TCA cycle, mevalonate pathway, terpenoid synthesis pathway, shikimate pathways, and ultimately with camptothecin biosynthesis, as revealed from the metabolic interactions from the KEGG pathway mapper (64 – 66). From the metabolic interaction and clustering analyses of the metabolic networks, several key interacting pathway components including antioxidant, carbohydrate metabolism, pentose phosphate pathway, oxidative phosphorylation, and translation regulation enzymatic systems were metabolically related to secondary metabolites especially camptothecin biosynthesis.

In conclusion, decreasing the expression of camptothecin productivity by fungi with storage and subculturing is the challenge that halts their further implementation to be an industrial platform for CPT production. The initial biosynthetic machinery of camptothecin by *A. terreus* was suppressed by about 50% by the seventh cultural generation; however, the productivity of camptothecin by the fungus has been completely restored upon addition of *F. elastica* microbiome. Restoring the camptothecin biosynthetic machinery of *A. terreus* ensures the microbial interactions releasing some signals that trigger the cryptic genes of camptothecin biosynthesis by *A. terreus*. To explore the attenuation/restoration of camptothecin biosynthetic machinery, differential proteomic and metabolomics analyses were conducted to the zero culture of *A. terreus*, seventh culture of *A. terreus*, and seventh culture with *F. elastica* microbiome. From the GO analyses, the molecular functions process ribosomal proteins, ATP binding proteins, metal ion binding, RNA binding proteins, GTP binding proteins, and GTPase activity were noticeably abolished by the seventh *A. terreus*, and complete restored with *F. elastica* microbiome. The highest differentially abundant proteins were Pleckstrin homology -domain-containing protein, mitochondrial peptidylprolyl *cis-trans* isomerase, and 60S ribosomal protein L6 that fluctuated with the subculturing of *A. terreus* with addition of *F. elastica* microbiome, with remarkable relation to camptothecin biosynthesis. Practically, the expression of *A. terreus*, PH domain-containing protein, and peptidylprolyl cis-trans isomerase was significantly increasingly responsive to microbiome of *F. elastica*, confirming the remodeling chromatin of *A. terreus* restoring the expression of camptothecin biosynthetic genes.

## Data Availability

All data generated during this study are included in this published article and its supplementary information files.

## SUPPLEMENTAL MATERIAL

Table S1 spectrum.02281-23-s0001.docx Peptide names and their percent coverage in zero culture, in 7th culture of *A. terreus*, and in response to amendment with *F. elastica* microbiome.

ASM does not own the copyrights to Supplemental Material that may be linked to, or accessed through, an article. The authors have granted ASM a non-exclusive, world-wide license to publish the Supplemental Material files. Please contact the corresponding author directly for reuse.
