# Supplementary material for: Proteomics and metabolomics analyses of camptothecin-producing Aspergillus terreus reveal the integration of PH domain-containing proteins and peptidylprolyl cis/trans isomerase in restoring the camptothecin biosynthesis
Source: Microbiol Spectr. 2023 Oct 19;11(6):e02281-23. doi: 10.1128/spectrum.02281-23 (PMC10714794; doi:10.1128/spectrum.02281-23)
Supplement: Table S1 — Peptide names and their percent coverage in zero culture, in 7th culture of A. terreus, and in response to amendment with F. elastica microbiome. [file spectrum.02281-23-s0001.docx]

Table S1: Peptides names and their coverage % in the zero culture, 7^th^ culture of A. terreus and in response to amendment with F. elastica microbiome

|  | A.terreus Zero | | A. terreus 7th Generation | | A. terreus+ F. elastica | |
| --- | --- | --- | --- | --- | --- | --- |
| No | Accession # | Coverage (%) | Accession # | Coverage (%) | Accession # | Coverage (%) |
| 1 | A0A017SQB6 | 33.82 | Q0CPK2 | 6.43 | Q0CTS7 | 10 |
| 2 | P51044 | 3.16 | Q0CNF9 | 33.5 | A0A0J5SKL9 | 2.78 |
| 3 | Q0CLA1 | 50.32 | A0A370TPX9 | 1.9 | A0A0F0INT0 | 11.36 |
| 4 | Q0CYI6 | 4.07 | Q0CU35 | 2.84 | A0A0L1J8M5 | 33.79 |
| 5 | Q5B135 | 14.38 | Q0CLA1 | 21.94 | Q0CCZ7 | 4.85 |
| 6 | A0A0F8U046 | 7.53 | Q0CU25 | 51.22 | Q0CCZ8 | 43.84 |
| 7 | A0A0F0INT0 | 11.36 | A0A1L9R5J9 | 8.74 | A0A2P2HUD4 | 4.37 |
| 8 | A0A0L1J8M5 | 7.59 | Q0CGV7 | 26.45 | Q0CLG2 | 7.03 |
| 9 | A0A017SRG4 | 7.42 | Q0CER7 | 15.34 | A0A2I2G2W0 | 19.23 |
| 10 | Q5ERH6 | 11.42 | Q0CAT1 | 1.99 | Q0CCS9 | 4.64 |
| 11 | Q2U5T5 | 2.25 | A0A0K8LB42 | 12.38 | Q0CTT4 | 18.08 |
| 12 | Q0CLG2 | 20.44 | A0A017SP17 | 3.52 | Q0CU57 | 34.85 |
| 13 | A0A0F0IHD8 | 3.75 | Q0CRJ6 | 21.56 | Q0CUA4 | 28.67 |
| 14 | Q0CLQ5 | 2.46 | Q4WHY9 | 6.34 | A0A0J5PVQ0 | 3.41 |
| 15 | Q0CLQ2 | 26.25 | A0A229XAQ1 | 1.6 | A0A318ZQ93 | 1.97 |
| 16 | A0A017S5R4 | 4.25 | O43099 | 5.95 | A0A0F0IAN9 | 7.32 |
| 17 | A0A0F0IB81 | 1.43 | Q0CCY0 | 23.01 | A0A0F8UZQ2 | 1.75 |
| 18 | Q0CLN3 | 6.26 | Q0D1R1 | 10 | A0A023SGN0 | 17.11 |
| 19 | Q0CLN4 | 10.55 | Q0C9B9 | 2.83 | P16928 | 1.49 |
| 20 | A0A0L1J6D6 | 7.89 | Q0D210 | 3.28 | Q0CBN8 | 7.84 |
| 21 | Q0CLL8 | 6.9 | Q0CYA7 | 3.9 | A0A0K8LFC7 | 33.82 |
| 22 | A0A0J5PJR5 | 7.91 | A0A2J5I777 | 3.6 | P20445 | 2.38 |
| 23 | Q0CLZ5 | 12.1 | Q0CTV1 | 21.01 | Q0C9W6 | 44.19 |
| 24 | Q0CLY1 | 8.59 | Q0CB11 | 5.91 | A0A017SRY4 | 4.95 |
| 25 | A0A254U1K7 | 12.44 | Q0CPM4 | 3.11 | A0A017SCR1 | 6.45 |
| 26 | A0A017SKQ8 | 21.68 | A0A370TGC1 | 2.39 | Q09036 | 5.63 |
| 27 | A0A0F0IAN9 | 7.32 | Q0CVY4 | 19.83 | Q0CT54 | 65.39 |
| 28 | Q0CM99 | 7.65 | Q4WGE2 | 1.48 | A0A0F8TZZ0 | 5.65 |
| 29 | A0A0J5PYK0 | 2.35 | A0A1S9DDI7 | 2.96 | A0A0F8UG87 | 3.18 |
| 30 | A0A317W4N1 | 3.79 | Q0CDC6 | 5.47 | A0A0F0I8H7 | 2.89 |
| 31 | Q0CM63 | 39.23 | A0A397GBQ7 | 8.4 | A0A1L9SK52 | 6.96 |
| 32 | Q0CLT3 | 1.31 | P85978 | 6.49 | P0C952 | 6.72 |
| 33 | Q0CKA9 | 2.07 | A0A229Z0B2 | 11.11 | Q0CKQ8 | 7.99 |
| 34 | A0A0F0IJ55 | 14.04 | Q0CWG3 | 27.46 | A0A0F8UWW7 | 13.85 |
| 35 | P16928 | 1.49 | Q0CYJ2 | 21.28 | Q0CJ60 | 20.3 |
| 36 | A0A0F8UV16 | 9.7 | Q0CFD3 | 9.94 | A0A017SIC6 | 14.13 |
| 37 | A0A0F0I936 | 10.64 | Q0CUA4 | 47.33 | B8XPJ9 | 8.15 |
| 38 | A0A0F8X1S4 | 16.3 | Q0CNS7 | 6.45 | D0VKF5 | 1.51 |
| 39 | A0A0F0IH70 | 1.36 | A0A0F0IRR4 | 10.08 | A0A017S1P3 | 2.4 |
| 40 | C1KIY8 | 5.95 | A0A017SNN2 | 3.51 | P85978 | 14.94 |
| 41 | A0A0F0I9X8 | 30.84 | Q0CWC4 | 1 | Q0CKY0 | 14.94 |
| 42 | A0A0F0IKG9 | 8.23 | Q0CNQ8 | 11.08 | Q0CKY1 | 25.07 |
| 43 | Q0CKD3 | 16.63 | Q0D0A2 | 1.91 | A0A2I6SS11 | 2.05 |
| 44 | Q0CKQ8 | 15.21 | A0A017SJL9 | 12.62 | H9BAU8 | 8.45 |
| 45 | Q0CL25 | 23.12 | Q0CX28 | 5.41 | A0A0F0IK84 | 15.58 |
| 46 | A0A0F0I8T7 | 3.39 | Q0CWQ9 | 25.47 | A0A1L9SMY4 | 3.09 |
| 47 | Q0CL04 | 7.8 | A0A0J5Q715 | 7.6 | Q0CAK5 | 4.3 |
| 48 | A0A017SC42 | 4.62 | I8A9J6 | 1.18 | Q0CRL8 | 8.81 |
| 49 | Q0CKY0 | 52.6 | Q0CQD0 | 3.51 | Q0CIX6 | 25 |
| 50 | A0A1L9SY53 | 2.04 | A0A0F0IQZ6 | 2.83 | A0A1L9SD22 | 3.05 |
| 51 | Q0CKY1 | 30.48 | Q0C7P9 | 6.98 | Q0CS84 | 8.26 |
| 52 | Q0CL92 | 27.97 | A0A395GPZ7 | 0.38 | A0A2J5I777 | 3.6 |
| 53 | Q0CKX4 | 1.54 | A0A017SM14 | 1.43 | Q0CB11 | 22.58 |
| 54 | Q0CL85 | 4.57 | Q0C9S3 | 23.76 | A0A017SBT5 | 5.52 |
| 55 | Q0CL83 | 29.51 | Q0C9S0 | 11.83 | A0A0F0I5V0 | 1.98 |
| 56 | A0A0J5PRI0 | 2.52 | P20445 | 2.38 | Q0CJN9 | 2.09 |
| 57 | A0A0F0I6W1 | 7.95 | Q0CFK0 | 5.46 | A0A2P2HCI3 | 3.22 |
| 58 | Q0CL64 | 20.3 | Q0CSD3 | 15.62 | A0A0J5PYS4 | 1.72 |
| 59 | H9BAU8 | 15.07 | A0A017SG56 | 3.31 | Q0CJK0 | 7.18 |
| 60 | Q0CKS3 | 6.47 | Q0C7L1 | 5.67 | A0A0F0IAN9 | 8.13 |
| 61 | A0A0F0IP66 | 7.48 | Q0C9P1 | 8.29 | Q0C9G6 | 2.44 |
| 62 | Q0CIX6 | 45.59 | A0A100IK75 | 2.32 | A0A017SJL9 | 12.62 |
| 63 | A0A117DWQ6 | 6.33 | Q0CK20 | 5.71 | Q0CJW4 | 0.48 |
| 64 | A1C5F0 | 13.9 | Q0CUF0 | 4.15 | A0A0F0IQZ6 | 2.83 |
| 65 | Q5DR85 | 13.43 | Q0D093 | 7.43 | A0A2J5HVJ6 | 9.78 |
| 66 | Q0CJA9 | 44.67 | Q0CKA9 | 3.92 | Q0CJV1 | 9.49 |
| 67 | A0A0F8U046 | 9.25 | P85978 | 8.44 | Q0C9S3 | 24.75 |
| 68 | A0A017SKQ8 | 19.58 | Q0D0I2 | 2.05 | Q0C9S0 | 29.77 |
| 69 | Q0CLU2 | 4.93 | A0A017S7S5 | 0 | Q0CBD1 | 75 |
| 70 | A0A017SSH2 | 4.05 | P85978 | 0 | A0A0F8TWR6 | 7.79 |
| 71 | Q0CJK0 | 11.96 | Q9HGY6 | 1.9 | A0A0L1IRP7 | 4.9 |
| 72 | A0A1L9WT41 | 2.11 | Q0D0H1 | 37.32 | Q0C9P1 | 22.8 |
| 73 | O74699 | 18.12 | A0A017SE33 | 2.87 | C8VFP7 | 91.3 |
| 74 | A0A317X2Y1 | 2.95 | Q0CMC2 | 59.77 | A0A0J5PPQ1 | 15.65 |
| 75 | Q0CK88 | 8.33 | Q0CUS1 | 6.82 | A0A2G7EMG1 | 1.88 |
| 76 | Q0CJW4 | 5.82 | Q4WGL5 | 2.66 | A0A1F7ZL68 | 2 |
| 77 | Q0CJV1 | 18.25 | Q0CE25 | 2.62 | Q0CQH7 | 3.75 |
| 78 | A0A017SCJ4 | 3.27 | Q0D0F9 | 34.86 | Q0C867 | 4.17 |
| 79 | A0A0L1IRP7 | 8.29 | Q0CMA6 | 1.65 | A0A0U5GQE6 | 6.09 |
| 80 | Q2U4K2 | 18.31 | A0A0F0IEU8 | 1.99 | A1C6S8 | 2.51 |
| 81 | A0A0L1ISH5 | 5.01 | Q0CUP4 | 22.47 | Q0CQE9 | 3.98 |
| 82 | A2RQB8 | 13.14 | Q0C9X7 | 5.61 | A0A017SQX4 | 4.57 |
| 83 | A0A0L1JD75 | 6.56 | Q0CFQ7 | 26.9 | A0A1L9SK52 | 8.26 |
| 84 | A0A0L1J5X8 | 20.28 | A0A0F8W8U8 | 20.3 | Q0CID8 | 14.55 |
| 85 | Q0CHW8 | 3.46 | Q0C9W6 | 4.65 | A0A017SM80 | 14.78 |
| 86 | P87256 | 3.86 | A0A1L9SK52 | 2.17 | A0A017SQL7 | 14.75 |
| 87 | Q0CHV8 | 4.03 | Q0CG11 | 10.08 | A0A0S7DL67 | 8.59 |
| 88 | A0A017SQX4 | 6.74 | Q0D128 | 22.58 | Q0CIA3 | 7.26 |
| 89 | A0A100I9K4 | 2.03 | Q0CA49 | 23.85 | A0A1F7ZS66 | 17.65 |
| 90 | Q0CIE0 | 35.94 | Q0CID8 | 29.49 | A0A0J5PYI6 | 1.91 |
| 91 | A0A1L9RRD1 | 5.24 | Q0CML4 | 8.38 | Q0C8K3 | 3.58 |
| 92 | A0A100INB6 | 9.41 | Q0CDZ5 | 15.82 | A0A0F0INT0 | 13.64 |
| 93 | Q0CIA1 | 13.64 | Q0CR35 | 18.75 | Q0C8D5 | 3.14 |
| 94 | Q0CIA3 | 51.71 | A0A017SED3 | 8.13 | A0A1L9P8Q4 | 8.8 |
| 95 | A0A1F7ZS66 | 14.71 | Q0CUZ7 | 6.32 | A0A0K8LJ44 | 8.7 |
| 96 | Q0CIJ2 | 38.86 | A0A0S7DL67 | 8.59 | A0A0J5PGY0 | 18.75 |
| 97 | A0A0J5SJ77 | 27.82 | Q0CIA1 | 9.47 | A0A0F0I598 | 6.76 |
| 98 | Q0CIG1 | 4.59 | Q0CIA3 | 27.35 | A0A0C5KR71 | 6.81 |
| 99 | Q5AWS6 | 1.82 | Q0CMI1 | 6.4 | A0A0J5PWN4 | 8 |
| 100 | Q0CIU3 | 8.66 | A0A0F0I277 | 2.94 | A0A0F8U541 | 2.47 |
| 101 | Q0CJ31 | 8.26 | P0C953 | 20.9 | Q0CGV7 | 12.9 |
| 102 | Q0CIP7 | 17.14 | A0A0K8L1F3 | 2.74 | A0A0N7H563 | 13.95 |
| 103 | Q0CPK7 | 8.1 | Q0CT54 | 5.46 | Q0CXW2 | 7.75 |
| 104 | Q0CPK2 | 14.93 | A0A0F0I277 | 3.82 | Q0CPF2 | 4.04 |
| 105 | Q0CGG7 | 2.03 | A0A397HU68 | 0 | A0A100IM22 | 0.93 |
| 106 | A0A0U5C6G6 | 6.33 | A0A017SHT3 | 14.29 | Q0CH50 | 5.84 |
| 107 | Q0CPI2 | 3.56 | B8N106 | 2.98 | Q0CPE0 | 54.55 |
| 108 | Q0CPH5 | 12.48 | A2RVC1 | 36.84 | A0A1E3BSA9 | 18.37 |
| 109 | A0A0L1JFI6 | 17.03 | A0A0F8UG87 | 3.18 | A0A017SA32 | 3.33 |
| 110 | A0A017SC26 | 7.33 | A0A2I2F4B1 | 17.81 | Q0CPD7 | 8.4 |
| 111 | Q0CPG5 | 16.62 | Q0D0J9 | 2.33 | A0A318Z9A4 | 3.71 |
| 112 | Q0D1G0 | 28.06 | A0A1L9SK52 | 2.17 | Q0CYA7 | 8.6 |
| 113 | Q0CPF2 | 9.12 | Q0CSR2 | 64.04 | A0A0F0I1V9 | 16.9 |
| 114 | Q0CPF5 | 4.73 | Q0CXK4 | 2.95 | A0A017SRI2 | 6.95 |
| 115 | Q0CPE7 | 30.77 | A0A2V5H525 | 3.94 | A0A0F0I099 | 4.45 |
| 116 | Q0D1E7 | 23.36 | Q0CKQ8 | 4.88 | A0A0F8WI96 | 4.75 |
| 117 | Q0D1E1 | 32.26 | A0A229XID6 | 2.13 | Q0CYG7 | 11.96 |
| 118 | Q0CPE0 | 70 | Q8NKA5 | 2.24 | A0A0J5SES8 | 7.5 |
| 119 | Q0D1S6 | 32.31 | A0A0F8UWW7 | 13.85 | A0A0F8V067 | 10.1 |
| 120 | Q0D231 | 13.03 | Q0CXH0 | 7.01 | A0A3A3A1A2 | 11.18 |
| 121 | Q0D1R1 | 24.67 | A0A0F8VEG6 | 2.38 | Q0CI14 | 26.09 |
| 122 | Q0CQ10 | 32.62 | Q0CIJ2 | 49.14 | A0A3A2ZBE6 | 2.44 |
| 123 | Q0D210 | 12.77 | Q0CKN5 | 8.2 | Q0CWW9 | 3.3 |
| 124 | A0A017SPJ0 | 9.09 | Q0CMR8 | 2.99 | Q0CX81 | 1.21 |
| 125 | Q0CQ04 | 10.24 | Q0C8H2 | 6.12 | Q0D0F9 | 8 |
| 126 | A0A017S3Y7 | 17.74 | Q0CJ60 | 27.52 | A0A318ZJV4 | 2.06 |
| 127 | Q0D1M1 | 13.04 | Q0C8G6 | 23.63 | A0A0F0IRN8 | 4.49 |
| 128 | A0A0F0I099 | 4.45 | A0A2I2G4W8 | 3.56 | A0A017S8B0 | 3.2 |
| 129 | Q0D1W5 | 29.09 | A0A100INB6 | 3.05 | G9BX80 | 16.77 |
| 130 | Q0CQ86 | 6.8 | A0A2I2G2W0 | 16.35 | A0A017SED3 | 8.13 |
| 131 | Q0CQ73 | 27.13 | A2RVC1 | 27.63 | A0A0F0I640 | 20.83 |
| 132 | Q0D1V3 | 4.75 | Q0D146 | 8.15 | A2RVC1 | 11.84 |
| 133 | Q0CPU7 | 17.24 | A0A017S1P3 | 2.4 | A1CMM5 | 1.17 |
| 134 | Q0CPT3 | 49.18 | Q0C8D5 | 3.14 | Q0CXH0 | 7.01 |
| 135 | A0A1L9S1H3 | 4.94 | Q0CY49 | 3.27 | Q0D170 | 10.12 |
| 136 | Q0CQD9 | 5.73 | Q0CVN0 | 12.37 | Q0D163 | 29.65 |
| 137 | A0A2I2FAZ1 | 2.44 | Q0CZU8 | 26.82 | Q0CXF2 | 14.93 |
| 138 | A0A017SDF7 | 5.69 | A0A0J5PTE1 | 2.04 | Q9HEY7 | 11.04 |
| 139 | A0A1L9SPK7 | 10.11 | A0A0J5SZV0 | 0.86 | Q0D146 | 8.15 |
| 140 | A0A017SFE7 | 6.6 | A0A0K8L1B2 | 9.78 | Q0CXS6 | 18.56 |
| 141 | A0A017S3N1 | 3.36 | Q0CTH6 | 15.96 | A0A0F0I2Z7 | 11.69 |
| 142 | A0A017S740 | 5.84 | A0A0J5PRI0 | 2.52 | A0A1L9WLB6 | 7.12 |
| 143 | A0A017SPI5 | 8.6 | Q0CMZ4 | 3.16 | A0A0U5GQE6 | 6.74 |
| 144 | A0A0J5SIY5 | 2.28 | Q4X136 | 4.57 | A0A319B789 | 1.16 |
| 145 | Q0D0I2 | 27.19 | Q0CL64 | 48.87 | A0A017RZD1 | 16.5 |
| 146 | Q0D0I3 | 6.53 | Q0CL48 | 6.36 | A0A017SK22 | 6.48 |
| 147 | A0A254TUY7 | 16.94 | A0A017SJL9 | 11.65 | A0A0F0I805 | 2.39 |
| 148 | A0A017S656 | 25.32 | Q0CEF4 | 9.19 | A0A0F8U1X4 | 4.38 |
| 149 | Q0D0F9 | 8 |  |  | Q0CF36 | 3.5 |
| 150 | A0A100IH14 | 10.57 |  |  | Q0CVS7 | 6.09 |
| 151 | A0A017SF88 | 10.46 |  |  | A0A0K8LB42 | 12.38 |
| 152 | Q0D0C0 | 3.94 |  |  | Q0CVR3 | 1.23 |
| 153 | Q0D128 | 18.28 |  |  | Q0CF08 | 31.19 |
| 154 | Q0D0Q3 | 22.26 |  |  | A0A0U5CF80 | 3.22 |
| 155 | Q0D0Q0 | 8.03 |  |  | A0A017SG56 | 5.25 |
| 156 | G9BX80 | 34.8 |  |  | Q0CNN1 | 13.89 |
| 157 | Q0CP06 | 6.45 |  |  | Q0CNL8 | 6.77 |
| 158 | Q0D0L5 | 38.71 |  |  | Q0CEZ7 | 2.4 |
| 159 | A0A1L9SK52 | 2.83 |  |  | P0C2C7 | 27.72 |
| 160 | A0A0F0I6D1 | 2.73 |  |  | A0A017SPJ0 | 12.12 |
| 161 | A0A0U5GDX9 | 4.8 |  |  | A0A017SCW9 | 4.76 |
| 162 | Q0D170 | 39.88 |  |  | Q0CNW9 | 1.19 |
| 163 | Q0CP71 | 13.8 |  |  | Q0CNW8 | 17.5 |
| 164 | A0A0L1IXM1 | 3.88 |  |  | A0A0F8UBD2 | 4.2 |
| 165 | Q0D163 | 34.07 |  |  | A0A1L9WIL4 | 13.97 |
| 166 | Q0CP61 | 12.62 |  |  | Q0CUU2 | 6.35 |
| 167 | Q0D148 | 30.94 |  |  | A0A017SK11 | 19.23 |
| 168 | Q0D146 | 8.15 |  |  | Q0D0A2 | 1.91 |
| 169 | Q0CPB9 | 34.53 |  |  | A0A017SM00 | 16.09 |
| 170 | Q0D1B2 | 1.89 |  |  | A0A017SG56 | 6.61 |
| 171 | A0A0L1IL21 | 34.38 |  |  | Q0CNY2 | 6.97 |
| 172 | A0A0F0IGZ4 | 6.88 |  |  | C8VM21 | 2.26 |
| 173 | A0A0F0I5V0 | 10.79 |  |  | Q0D093 | 7.43 |
| 174 | A0A0L1IXM1 | 4.71 |  |  | Q0C9S9 | 15 |
| 175 | Q0D1A7 | 16.81 |  |  | A0A0J5PUM3 | 6.69 |
| 176 | A0A0K8L8B4 | 21.89 |  |  | Q0CE18 | 11.11 |
| 177 | A0A124BVS7 | 4.48 |  |  | Q0CUP4 | 6.46 |
| 178 | A0A0F0I302 | 9.09 |  |  | A0A0J5SUE8 | 19.57 |
| 179 | A0A0K8LB42 | 14.29 |  |  | A0A0F0IE61 | 4.1 |
| 180 | A0A2I2FB40 | 3.14 |  |  | Q0CE88 | 9.56 |
| 181 | A0A0L1IQV5 | 3.83 |  |  | A0A017S287 | 2.66 |
| 182 | Q0CNN1 | 17.78 |  |  | A0A0F8VKQ5 | 5.61 |
| 183 | A0A370BVT3 | 4.63 |  |  | A0A124BWZ2 | 3.33 |
| 184 | A0A1R3RGQ8 | 17.5 |  |  | A0A3A2ZPD4 | 3.33 |
| 185 | Q0CNK7 | 2.97 |  |  | Q0CMR4 | 9.05 |
| 186 | Q0CNI8 | 4.23 |  |  | A0A0F8V067 | 16.16 |
| 187 | Q0CNW9 | 5.34 |  |  | A0A0J5PUA7 | 1.48 |
| 188 | Q0CNW8 | 10 |  |  | Q0CVN0 | 5.38 |
| 189 | A0A2L0V3A4 | 44.06 |  |  | Q9P443 | 5.06 |
| 190 | Q0D076 | 8.46 |  |  | A0A0F8X691 | 22 |
| 191 | Q0D077 | 23.33 |  |  | A0A0K8L1B2 | 9.78 |
| 192 | Q0CNV1 | 23.55 |  |  | A0A017SQL8 | 8.15 |
| 193 | Q0CNS7 | 11.29 |  |  | A0A017SNU3 | 3.55 |
| 194 | A0A0F0IRR4 | 10.08 |  |  | A0A100IQ27 | 2.25 |
| 195 | A0A100IS99 | 11.28 |  |  | Q0CEF4 | 6.01 |
| 196 | Q0CNS4 | 28.78 |  |  | A0A017SHP9 | 19.51 |
| 197 | A0A0F8U2W7 | 10 |  |  |  |  |
| 198 | Q0D034 | 1.25 |  |  |  |  |
| 199 | Q0D035 | 38.31 |  |  |  |  |
| 200 | Q0D033 | 11.92 |  |  |  |  |
| 201 | Q0CNQ8 | 38.79 |  |  |  |  |
| 202 | Q0D025 | 4.38 |  |  |  |  |
| 203 | A0A117E032 | 0.66 |  |  |  |  |
| 204 | Q0D0A2 | 20.13 |  |  |  |  |
| 205 | A0A0U5FWV6 | 3.48 |  |  |  |  |
| 206 | A0A017SM00 | 29.89 |  |  |  |  |
| 207 | A0A370TM96 | 9.27 |  |  |  |  |
| 208 | A0A1L9TIV1 | 1.95 |  |  |  |  |
| 209 | Q0CNY2 | 44.28 |  |  |  |  |
| 210 | A0A017SF88 | 9.8 |  |  |  |  |
| 211 | Q0D093 | 26.35 |  |  |  |  |
| 212 | A0A100IUG9 | 12.31 |  |  |  |  |
| 213 | Q0CMA9 | 6.6 |  |  |  |  |
| 214 | Q0CMA7 | 20.82 |  |  |  |  |
| 215 | A0A100IR94 | 1.89 |  |  |  |  |
| 216 | Q4WAR0 | 1.77 |  |  |  |  |
| 217 | P60204 | 8.72 |  |  |  |  |
| 218 | A0A0K8KZI7 | 2.22 |  |  |  |  |
| 219 | A0A017SQX4 | 1.96 |  |  |  |  |
| 220 | Q0CML4 | 3.55 |  |  |  |  |
| 221 | A0A0F0IE61 | 4.1 |  |  |  |  |
| 222 | A0A124BY92 | 2.52 |  |  |  |  |
| 223 | Q0CMH3 | 21.91 |  |  |  |  |
| 224 | A2RVC1 | 44.74 |  |  |  |  |
| 225 | Q0CN60 | 18.45 |  |  |  |  |
| 226 | Q0CMT8 | 4.52 |  |  |  |  |
| 227 | A0A0J5PIC6 | 8.61 |  |  |  |  |
| 228 | Q0CMS4 | 23.33 |  |  |  |  |
| 229 | Q0CMR4 | 27.77 |  |  |  |  |
| 230 | A0A0F8V067 | 16.16 |  |  |  |  |
| 231 | Q9P443 | 6.23 |  |  |  |  |
| 232 | A0A0F0IGZ4 | 15.34 |  |  |  |  |
| 233 | Q0CMZ4 | 13.59 |  |  |  |  |
| 234 | A0A0F8TXS3 | 0.59 |  |  |  |  |
| 235 | A0A317WSH8 | 25.27 |  |  |  |  |
| 236 | Q0CTS7 | 18.33 |  |  |  |  |
| 237 | A0A1L9WMG7 | 4.53 |  |  |  |  |
| 238 | Q0CTR9 | 23.83 |  |  |  |  |
| 239 | Q0CU34 | 5.36 |  |  |  |  |
| 240 | Q0CU35 | 2.84 |  |  |  |  |
| 241 | Q0CU25 | 18.29 |  |  |  |  |
| 242 | A0A0U4Z8T0 | 6.47 |  |  |  |  |
| 243 | Q0CU95 | 13 |  |  |  |  |
| 244 | P0C953 | 23.88 |  |  |  |  |
| 245 | A0A0F0INT0 | 10.23 |  |  |  |  |
| 246 | Q0CTV1 | 19.89 |  |  |  |  |
| 247 | A0A017SPT2 | 1.4 |  |  |  |  |
| 248 | Q0CTT4 | 40.59 |  |  |  |  |
| 249 | Q0CU57 | 21.21 |  |  |  |  |
| 250 | Q0CIB5 | 14.47 |  |  |  |  |
| 251 | Q0CUA4 | 38 |  |  |  |  |
| 252 | P59769 | 1.79 |  |  |  |  |
| 253 | A0A0F0ILY1 | 8.09 |  |  |  |  |
| 254 | A0A017S083 | 7.19 |  |  |  |  |
| 255 | P35211 | 9.18 |  |  |  |  |
| 256 | A0A0J5PHR9 | 9.32 |  |  |  |  |
| 257 | Q0CT18 | 10.28 |  |  |  |  |
| 258 | A0A017S473 | 6.2 |  |  |  |  |
| 259 | A0A0K8LFC7 | 23.53 |  |  |  |  |
| 260 | Q0CSN1 | 9.03 |  |  |  |  |
| 261 | Q0CSM9 | 50.46 |  |  |  |  |
| 262 | Q0CSM3 | 26.53 |  |  |  |  |
| 263 | Q0CSL9 | 5.8 |  |  |  |  |
| 264 | Q0CSK4 | 31.51 |  |  |  |  |
| 265 | A0A1L9PX57 | 9.26 |  |  |  |  |
| 266 | Q0CSV2 | 7.06 |  |  |  |  |
| 267 | A0A017SFE3 | 2.27 |  |  |  |  |
| 268 | Q0CT54 | 70.86 |  |  |  |  |
| 269 | A0A1L9SK52 | 11.3 |  |  |  |  |
| 270 | Q0CTC0 | 39.54 |  |  |  |  |
| 271 | A0A0F0I3U1 | 51.46 |  |  |  |  |
| 272 | Q0CTB3 | 1.26 |  |  |  |  |
| 273 | A0A0F8UWB1 | 2.07 |  |  |  |  |
| 274 | A0A1S9DXS4 | 22.12 |  |  |  |  |
| 275 | A0A2I2FV13 | 6.99 |  |  |  |  |
| 276 | A0A017S1P3 | 2.4 |  |  |  |  |
| 277 | Q0CTK8 | 8.99 |  |  |  |  |
| 278 | G3K507 | 1.96 |  |  |  |  |
| 279 | A0A0F0I2N8 | 6.17 |  |  |  |  |
| 280 | A0A2I6SS11 | 18.81 |  |  |  |  |
| 281 | Q0CTH6 | 22.63 |  |  |  |  |
| 282 | Q0CTG5 | 10.58 |  |  |  |  |
| 283 | A0A0J5PNE9 | 7.94 |  |  |  |  |
| 284 | A1CAE1 | 11.72 |  |  |  |  |
| 285 | Q0CRL8 | 7.12 |  |  |  |  |
| 286 | Q0CRJ6 | 20.63 |  |  |  |  |
| 287 | A0A1E3BJM0 | 28.04 |  |  |  |  |
| 288 | A0A317UXD3 | 6.11 |  |  |  |  |
| 289 | Q0CRQ0 | 32.6 |  |  |  |  |
| 290 | Q0CS21 | 23.43 |  |  |  |  |
| 291 | Q0CS18 | 8.82 |  |  |  |  |
| 292 | Q9P443 | 3.89 |  |  |  |  |
| 293 | A0A017SKQ8 | 20.98 |  |  |  |  |
| 294 | A0A017S0Z0 | 11.31 |  |  |  |  |
| 295 | A0A017SCX7 | 7.95 |  |  |  |  |
| 296 | Q0CSH1 | 50.39 |  |  |  |  |
| 297 | P52718 | 2.07 |  |  |  |  |
| 298 | Q0CSG9 | 12.82 |  |  |  |  |
| 299 | Q0CSE6 | 2.77 |  |  |  |  |
| 300 | Q0CSD2 | 38.71 |  |  |  |  |
| 301 | A0A017S0R4 | 2.68 |  |  |  |  |
| 302 | Q0CSB5 | 4.01 |  |  |  |  |
| 303 | Q0CQK9 | 18.43 |  |  |  |  |
| 304 | Q0CQK5 | 24.09 |  |  |  |  |
| 305 | A0A0F0INT0 | 17.05 |  |  |  |  |
| 306 | Q0CQJ4 | 13.04 |  |  |  |  |
| 307 | Q0CQI3 | 4.22 |  |  |  |  |
| 308 | Q0CQH7 | 3.75 |  |  |  |  |
| 309 | A0A100INE3 | 17.42 |  |  |  |  |
| 310 | Q0CQE9 | 15.92 |  |  |  |  |
| 311 | A0A1E3BQ56 | 1.65 |  |  |  |  |
| 312 | A0A0J5SIW4 | 8.11 |  |  |  |  |
| 313 | A0A0J5PN39 | 2.69 |  |  |  |  |
| 314 | Q0CR48 | 5.75 |  |  |  |  |
| 315 | Q0CR35 | 6.25 |  |  |  |  |
| 316 | Q0CR38 | 16.4 |  |  |  |  |
| 317 | Q0CQQ4 | 18.14 |  |  |  |  |
| 318 | Q0CQZ2 | 13.23 |  |  |  |  |
| 319 | A0A1L9PCM0 | 16.88 |  |  |  |  |
| 320 | Q0CR99 | 1.75 |  |  |  |  |
| 321 | Q0CR95 | 5.58 |  |  |  |  |
| 322 | F5BBE8 | 6.22 |  |  |  |  |
| 323 | Q0CQW4 | 36.12 |  |  |  |  |
| 324 | Q0CR77 | 27.22 |  |  |  |  |
| 325 | Q0CRD8 | 36.99 |  |  |  |  |
| 326 | Q0CRC8 | 6.05 |  |  |  |  |
| 327 | Q0CRB8 | 24.43 |  |  |  |  |
| 328 | A0A2I2GH48 | 10.91 |  |  |  |  |
| 329 | A0A0F8U541 | 2.47 |  |  |  |  |
| 330 | Q0CY94 | 60 |  |  |  |  |
| 331 | Q0CXW2 | 27.46 |  |  |  |  |
| 332 | Q0CXV8 | 24.22 |  |  |  |  |
| 333 | A0A1W5SKT4 | 3.37 |  |  |  |  |
| 334 | A0A017SRG4 | 10.16 |  |  |  |  |
| 335 | A0A318Z9A4 | 3.71 |  |  |  |  |
| 336 | A0A100I8Y2 | 3.21 |  |  |  |  |
| 337 | Q0CYD9 | 10.08 |  |  |  |  |
| 338 | A0A254TWC8 | 7.53 |  |  |  |  |
| 339 | A0A0F8UPK7 | 4.48 |  |  |  |  |
| 340 | Q0CYA7 | 18.82 |  |  |  |  |
| 341 | A0A0F0IF51 | 2.51 |  |  |  |  |
| 342 | A0A0F8W4P4 | 12.15 |  |  |  |  |
| 343 | A0A0F8V198 | 4.18 |  |  |  |  |
| 344 | A0A0J5PWD0 | 4.09 |  |  |  |  |
| 345 | Q0CYJ2 | 30.5 |  |  |  |  |
| 346 | Q0CYG7 | 31.52 |  |  |  |  |
| 347 | A0A317WRJ7 | 21.24 |  |  |  |  |
| 348 | Q0C7P6 | 5.14 |  |  |  |  |
| 349 | Q0CYR1 | 23.44 |  |  |  |  |
| 350 | A0A017SRI2 | 8.88 |  |  |  |  |
| 351 | C8VTE9 | 12.16 |  |  |  |  |
| 352 | A0A017SRG4 | 3.13 |  |  |  |  |
| 353 | Q0C7L1 | 5.33 |  |  |  |  |
| 354 | Q0C7L3 | 3.6 |  |  |  |  |
| 355 | A0A317VX47 | 2.43 |  |  |  |  |
| 356 | Q0CWX1 | 8.42 |  |  |  |  |
| 357 | Q0CWX0 | 4.34 |  |  |  |  |
| 358 | Q0CWW9 | 6.6 |  |  |  |  |
| 359 | Q4WGL5 | 2.66 |  |  |  |  |
| 360 | A0A0F0I7Y6 | 1.27 |  |  |  |  |
| 361 | A0A0F0IRN8 | 4.49 |  |  |  |  |
| 362 | A0A1F8AHF9 | 5.31 |  |  |  |  |
| 363 | Q0CWU3 | 6.22 |  |  |  |  |
| 364 | A0A017S0R4 | 8.39 |  |  |  |  |
| 365 | Q0CX54 | 30.08 |  |  |  |  |
| 366 | Q0CX49 | 2.92 |  |  |  |  |
| 367 | Q0CWR8 | 9.6 |  |  |  |  |
| 368 | A0A100I8F8 | 15.32 |  |  |  |  |
| 369 | Q0CXB1 | 1.42 |  |  |  |  |
| 370 | Q0CXA9 | 4.69 |  |  |  |  |
| 371 | Q0CXA8 | 6.46 |  |  |  |  |
| 372 | A0A100ISE5 | 11.58 |  |  |  |  |
| 373 | A0A0F0IC94 | 10.89 |  |  |  |  |
| 374 | A0A229WNB8 | 2.26 |  |  |  |  |
| 375 | Q0CXK3 | 3.13 |  |  |  |  |
| 376 | A0A2I2GIH3 | 11.16 |  |  |  |  |
| 377 | Q0C9U7 | 5.08 |  |  |  |  |
| 378 | Q0CXJ0 | 26.89 |  |  |  |  |
| 379 | A0A319DVC1 | 3.72 |  |  |  |  |
| 380 | Q0CXH0 | 26.75 |  |  |  |  |
| 381 | Q0CXF2 | 40.63 |  |  |  |  |
| 382 | Q0CXD0 | 12.63 |  |  |  |  |
| 383 | Q0CY49 | 3.27 |  |  |  |  |
| 384 | Q0CXS6 | 40.21 |  |  |  |  |
| 385 | Q0CXS1 | 16.18 |  |  |  |  |
| 386 | A0A0F8URI5 | 1.69 |  |  |  |  |
| 387 | Q0CXR4 | 7.64 |  |  |  |  |
| 388 | A0A1B0UHJ4 | 7.78 |  |  |  |  |
| 389 | A0A0F0I2Z7 | 9.09 |  |  |  |  |
| 390 | Q0CY16 | 29.03 |  |  |  |  |
| 391 | Q0CY12 | 3.12 |  |  |  |  |
| 392 | A0A0F0IJE6 | 4.04 |  |  |  |  |
| 393 | Q0CXN7 | 2.95 |  |  |  |  |
| 394 | A0A0L1IY77 | 12.42 |  |  |  |  |
| 395 | H6W8H5 | 16.89 |  |  |  |  |
| 396 | Q0CW86 | 14.44 |  |  |  |  |
| 397 | Q0CVV8 | 8.65 |  |  |  |  |
| 398 | A0A017S1P3 | 2.64 |  |  |  |  |
| 399 | A1CBG4 | 9.39 |  |  |  |  |
| 400 | Q0CVT0 | 3.7 |  |  |  |  |
| 401 | Q0CVS7 | 4.99 |  |  |  |  |
| 402 | Q0CVR5 | 1.92 |  |  |  |  |
| 403 | Q0CVR3 | 10.53 |  |  |  |  |
| 404 | Q0CVQ3 | 10.53 |  |  |  |  |
| 405 | A0A017SR81 | 6.67 |  |  |  |  |
| 406 | A0A017SPJ0 | 10.1 |  |  |  |  |
| 407 | Q0CVY4 | 25.67 |  |  |  |  |
| 408 | O93918 | 1.09 |  |  |  |  |
| 409 | Q0CW98 | 15.22 |  |  |  |  |
| 410 | A0A0J5PYT4 | 2.02 |  |  |  |  |
| 411 | Q0CWH3 | 8.72 |  |  |  |  |
| 412 | Q0CWE4 | 1.97 |  |  |  |  |
| 413 | A0A0F0HXQ0 | 7.28 |  |  |  |  |
| 414 | Q0CWD5 | 3.57 |  |  |  |  |
| 415 | Q0CUU2 | 19.05 |  |  |  |  |
| 416 | A0A017SGX6 | 3.6 |  |  |  |  |
| 417 | A0A2I1D9A7 | 20.11 |  |  |  |  |
| 418 | Q4WX89 | 5.19 |  |  |  |  |
| 419 | Q0CWA3 | 3.43 |  |  |  |  |
| 420 | Q0CWQ9 | 9.43 |  |  |  |  |
| 421 | Q0CWQ2 | 2.83 |  |  |  |  |
| 422 | P59769 | 1.93 |  |  |  |  |
| 423 | Q0CX14 | 3.13 |  |  |  |  |
| 424 | Q0CWN6 | 3.36 |  |  |  |  |
| 425 | A0A1L9WW95 | 12.96 |  |  |  |  |
| 426 | Q0CWI8 | 57.14 |  |  |  |  |
| 427 | Q0CUS1 | 12.34 |  |  |  |  |
| 428 | Q0CV39 | 7.84 |  |  |  |  |
| 429 | A0A0J5PFZ8 | 9.54 |  |  |  |  |
| 430 | A0A0F0IJJ4 | 11.49 |  |  |  |  |
| 431 | Q0CUQ8 | 19.23 |  |  |  |  |
| 432 | A0A0F0IRE1 | 2.15 |  |  |  |  |
| 433 | Q0CUP4 | 19.66 |  |  |  |  |
| 434 | A0A1F7ZPA0 | 41.33 |  |  |  |  |
| 435 | A0A2I1CIE0 | 4.68 |  |  |  |  |
| 436 | A0A0J5SUE8 | 19.57 |  |  |  |  |
| 437 | A0A0J5PXZ4 | 2.04 |  |  |  |  |
| 438 | Q874I0 | 2.99 |  |  |  |  |
| 439 | Q0CUZ7 | 25.86 |  |  |  |  |
| 440 | A0A0J5PU66 | 6.14 |  |  |  |  |
| 441 | A0A2I1DA85 | 7.38 |  |  |  |  |
| 442 | Q0CUY0 | 10.78 |  |  |  |  |
| 443 | A0A0J5PKX4 | 6.02 |  |  |  |  |
| 444 | A0A0L1JFX7 | 16.12 |  |  |  |  |
| 445 | A0A229XID6 | 2.13 |  |  |  |  |
| 446 | A0A017SRE1 | 8.76 |  |  |  |  |
| 447 | A0A1E3BPZ6 | 2.54 |  |  |  |  |
| 448 | P60204 | 10.74 |  |  |  |  |
| 449 | Q0CVN0 | 25.27 |  |  |  |  |
| 450 | A0A2V5HJ07 | 1.94 |  |  |  |  |
| 451 | A0A017SPI5 | 8.6 |  |  |  |  |
| 452 | Q0CR27 | 8.4 |  |  |  |  |
| 453 | A0A017SQL8 | 5.43 |  |  |  |  |
| 454 | Q0CVK8 | 46.75 |  |  |  |  |
| 455 | Q0CVJ6 | 5.04 |  |  |  |  |
| 456 | A0A0J5PJH7 | 3.32 |  |  |  |  |
| 457 | A0A0J5PM76 | 3.29 |  |  |  |  |
| 458 | A0A0J5PNI8 | 7.94 |  |  |  |  |
| 459 | A0A0F0IFT3 | 4.85 |  |  |  |  |
| 460 | P60204 | 7.38 |  |  |  |  |
| 461 | A0A0F0IEG8 | 2.71 |  |  |  |  |
| 462 | A0A017S3V8 | 4.11 |  |  |  |  |
| 463 | A0A017S3F0 | 15.33 |  |  |  |  |
| 464 | A0A0J5PVQ0 | 11.36 |  |  |  |  |
| 465 | Q4W1X2 | 1.96 |  |  |  |  |
| 466 | A0A117DWQ6 | 14.56 |  |  |  |  |
| 467 | A0A254U7Z3 | 2.57 |  |  |  |  |
| 468 | A0A1U8QEN9 | 9.32 |  |  |  |  |
| 469 | A0A1L9SG51 | 14.93 |  |  |  |  |
| 470 | A0A0F8U8S7 | 22.31 |  |  |  |  |
| 471 | A0A0U5GP80 | 1.68 |  |  |  |  |
| 472 | A0A318ZI11 | 1.32 |  |  |  |  |
| 473 | B8XPN5 | 45.65 |  |  |  |  |
| 474 | A0A017S964 | 8.7 |  |  |  |  |
| 475 | A0A2V5IA26 | 5.98 |  |  |  |  |
| 476 | Q0C9X7 | 18.69 |  |  |  |  |
| 477 | Q0C9W6 | 68.02 |  |  |  |  |
| 478 | A0A317VT09 | 1.35 |  |  |  |  |
| 479 | Q09036 | 5.63 |  |  |  |  |
| 480 | A0A2G7FVZ7 | 8.79 |  |  |  |  |
| 481 | A0A0J5Q4L0 | 10.34 |  |  |  |  |
| 482 | A0A0F8W4P4 | 10.93 |  |  |  |  |
| 483 | A0A0F0I326 | 7.84 |  |  |  |  |
| 484 | A1CMX4 | 2.92 |  |  |  |  |
| 485 | A0A017SKI1 | 5.76 |  |  |  |  |
| 486 | A0A0F0IEB6 | 4.83 |  |  |  |  |
| 487 | A0A317X738 | 6.04 |  |  |  |  |
| 488 | A0A1L9TPG6 | 10.95 |  |  |  |  |
| 489 | A0A0J5SIW4 | 11.71 |  |  |  |  |
| 490 | A0A0J5Q863 | 5.16 |  |  |  |  |
| 491 | Q0C8Z0 | 2.72 |  |  |  |  |
| 492 | Q0C8X9 | 18.55 |  |  |  |  |
| 493 | Q0CVA9 | 2.29 |  |  |  |  |
| 494 | Q0C987 | 7.43 |  |  |  |  |
| 495 | Q0C982 | 10.3 |  |  |  |  |
| 496 | Q0C8V9 | 11.07 |  |  |  |  |
| 497 | Q0C9D2 | 1.27 |  |  |  |  |
| 498 | Q0C9B9 | 7.42 |  |  |  |  |
| 499 | Q0C9B7 | 25.68 |  |  |  |  |
| 500 | A0A0F0IDE6 | 1.59 |  |  |  |  |
| 501 | Q9C1M3 | 6.6 |  |  |  |  |
| 502 | A0A2G7FKS6 | 1 |  |  |  |  |
| 503 | A0A0F8U0N7 | 4.67 |  |  |  |  |
| 504 | A0A2I2F654 | 7.74 |  |  |  |  |
| 505 | Q0C9G6 | 14.23 |  |  |  |  |
| 506 | A0A0S7DEP4 | 15.55 |  |  |  |  |
| 507 | A2RVC1 | 11.84 |  |  |  |  |
| 508 | A0A100IH14 | 8.94 |  |  |  |  |
| 509 | A0A1F8A338 | 1.43 |  |  |  |  |
| 510 | Q0C9S3 | 42.57 |  |  |  |  |
| 511 | Q0C9S0 | 69.08 |  |  |  |  |
| 512 | Q0C9P1 | 29.53 |  |  |  |  |
| 513 | A0A117DVB7 | 7.02 |  |  |  |  |
| 514 | A0A1S9DH82 | 2.05 |  |  |  |  |
| 515 | A0A017SRY4 | 19.31 |  |  |  |  |
| 516 | Q0C7Z0 | 16.11 |  |  |  |  |
| 517 | Q0C7Y0 | 12.64 |  |  |  |  |
| 518 | Q0C895 | 10.49 |  |  |  |  |
| 519 | Q0C896 | 8.71 |  |  |  |  |
| 520 | Q0C897 | 18.99 |  |  |  |  |
| 521 | A0A2I2G211 | 13.33 |  |  |  |  |
| 522 | C8VAU8 | 8.09 |  |  |  |  |
| 523 | A0A1S9DDI7 | 6.18 |  |  |  |  |
| 524 | A0A2I1CVP4 | 1.04 |  |  |  |  |
| 525 | Q0C867 | 6.94 |  |  |  |  |
| 526 | Q0C869 | 2.72 |  |  |  |  |
| 527 | A0A0F0IB08 | 9.68 |  |  |  |  |
| 528 | Q9HEY7 | 14.94 |  |  |  |  |
| 529 | Q0C8C7 | 14.29 |  |  |  |  |
| 530 | A0A0F8X027 | 10.34 |  |  |  |  |
| 531 | A0A0F0I6I4 | 2.91 |  |  |  |  |
| 532 | Q0C8K3 | 9.07 |  |  |  |  |
| 533 | Q0C8K4 | 5.42 |  |  |  |  |
| 534 | A0A0J5Q8D5 | 3.15 |  |  |  |  |
| 535 | Q0CZK5 | 28.08 |  |  |  |  |
| 536 | Q0CZK6 | 16.39 |  |  |  |  |
| 537 | Q0C8H2 | 10.61 |  |  |  |  |
| 538 | Q0C8G6 | 10.96 |  |  |  |  |
| 539 | Q0C8D5 | 10.91 |  |  |  |  |
| 540 | Q0CZU8 | 19.07 |  |  |  |  |
| 541 | A0A017S1P3 | 2.64 |  |  |  |  |
| 542 | Q0C928 | 7.45 |  |  |  |  |
| 543 | A0A0J5PL40 | 7.96 |  |  |  |  |
| 544 | A0A0F8TXI3 | 11.21 |  |  |  |  |
| 545 | A0A0F0I598 | 6.76 |  |  |  |  |
| 546 | A0A0U5GTE1 | 6.44 |  |  |  |  |
| 547 | A0A0S7DXQ0 | 9.77 |  |  |  |  |
| 548 | A0A0F8UZF3 | 2.46 |  |  |  |  |
| 549 | A0A0F0I5L6 | 9.68 |  |  |  |  |
| 550 | A0A2I2FZ67 | 14.14 |  |  |  |  |
| 551 | A0A0K8LJ44 | 6.52 |  |  |  |  |
| 552 | A0A017SRH2 | 12.61 |  |  |  |  |
| 553 | A0A1L9VJQ3 | 2.31 |  |  |  |  |
| 554 | A0A017S7N4 | 2.5 |  |  |  |  |
| 555 | A0A0J5PKH1 | 1.31 |  |  |  |  |
| 556 | A0A124BY38 | 18.37 |  |  |  |  |
| 557 | D3GAN9 | 6.58 |  |  |  |  |
| 558 | A0A0K8L6S8 | 0.68 |  |  |  |  |
| 559 | A0A318ZBR3 | 12.24 |  |  |  |  |
| 560 | A0A0J5PWK7 | 10.4 |  |  |  |  |
| 561 | A0A0J5Q5P1 | 5.03 |  |  |  |  |
| 562 | A0A0J5PSD7 | 4.92 |  |  |  |  |
| 563 | A0A100IPF9 | 7.19 |  |  |  |  |
| 564 | Q5B135 | 3.42 |  |  |  |  |
| 565 | A0A0F0I6W1 | 6.62 |  |  |  |  |
| 566 | A0A1L9R5J9 | 8.74 |  |  |  |  |
| 567 | A0A0F8W398 | 8.5 |  |  |  |  |
| 568 | A0A117E1W4 | 12.57 |  |  |  |  |
| 569 | P0C2C8 | 46.82 |  |  |  |  |
| 570 | P0C2C7 | 27.72 |  |  |  |  |
| 571 | A0A017SHC6 | 8.81 |  |  |  |  |
| 572 | A0A0J5SJ77 | 27.44 |  |  |  |  |
| 573 | P85978 | 6.49 |  |  |  |  |
| 574 | A0A0F0IKS0 | 15.97 |  |  |  |  |
| 575 | A0A2I1DGK4 | 6.09 |  |  |  |  |
| 576 | A0A0F0I9E1 | 8.05 |  |  |  |  |
| 577 | H9BAU8 | 13.01 |  |  |  |  |
| 578 | Q5AWS6 | 6.32 |  |  |  |  |
| 579 | A0A017SEI6 | 1.58 |  |  |  |  |
| 580 | A0A0F0I6W1 | 11.26 |  |  |  |  |
| 581 | Q9Y8A8 | 9.68 |  |  |  |  |
| 582 | A0A0D9MUA2 | 3.67 |  |  |  |  |
| 583 | A0A0F0I619 | 3.78 |  |  |  |  |
| 584 | A0A0F0IJD4 | 3.96 |  |  |  |  |
| 585 | A0A0L1IPU7 | 12.36 |  |  |  |  |
| 586 | A0A1R3RZ41 | 22.58 |  |  |  |  |
| 587 | A0A229XII5 | 3.73 |  |  |  |  |
| 588 | A0A0F0IKG9 | 11.26 |  |  |  |  |
| 589 | A0A0J5Q3C3 | 9.73 |  |  |  |  |
| 590 | A0A0F8TXI3 | 14.95 |  |  |  |  |
| 591 | A0A100INV0 | 9.15 |  |  |  |  |
| 592 | A0A1L9TGC4 | 13.01 |  |  |  |  |
| 593 | A0A0K8L1B2 | 9.78 |  |  |  |  |
| 594 | A0A0S7DRR6 | 1.33 |  |  |  |  |
| 595 | P40292 | 6.52 |  |  |  |  |
| 596 | Q0CD26 | 33.02 |  |  |  |  |
| 597 | A0A0F0ICR1 | 1.59 |  |  |  |  |
| 598 | Q0CCP9 | 34.38 |  |  |  |  |
| 599 | Q0CD12 | 28.22 |  |  |  |  |
| 600 | A0A0J5SSL7 | 8.43 |  |  |  |  |
| 601 | Q0CD08 | 11.98 |  |  |  |  |
| 602 | A0A0J5Q863 | 4.79 |  |  |  |  |
| 603 | A0A0F0II87 | 4.25 |  |  |  |  |
| 604 | Q0CCZ8 | 11.44 |  |  |  |  |
| 605 | A0A0F0INT0 | 14.77 |  |  |  |  |
| 606 | Q0CCY0 | 39.82 |  |  |  |  |
| 607 | Q0CD97 | 19.59 |  |  |  |  |
| 608 | A0A319E884 | 1.43 |  |  |  |  |
| 609 | Q0CCS9 | 26.38 |  |  |  |  |
| 610 | A0A017SGN7 | 2.2 |  |  |  |  |
| 611 | Q0CCR3 | 41.42 |  |  |  |  |
| 612 | Q0CDB7 | 4.61 |  |  |  |  |
| 613 | A0A0F8W1Z7 | 1.13 |  |  |  |  |
| 614 | Q0CIB5 | 27.63 |  |  |  |  |
| 615 | A0A017SCR8 | 7.59 |  |  |  |  |
| 616 | P56205 | 7.21 |  |  |  |  |
| 617 | A0A017SJY2 | 3.86 |  |  |  |  |
| 618 | A0A124BWF4 | 1.25 |  |  |  |  |
| 619 | A0A317XBV4 | 22.67 |  |  |  |  |
| 620 | A0A2I2FFA7 | 6.62 |  |  |  |  |
| 621 | A0A017SQ96 | 35.63 |  |  |  |  |
| 622 | A0A0F0IH70 | 1.13 |  |  |  |  |
| 623 | A0A0L1IZL1 | 5.67 |  |  |  |  |
| 624 | Q0CBP0 | 2.3 |  |  |  |  |
| 625 | A0A0F0I6Z6 | 14.51 |  |  |  |  |
| 626 | Q0CBN8 | 19.92 |  |  |  |  |
| 627 | Q0CBN9 | 8.28 |  |  |  |  |
| 628 | Q5ERH6 | 5.12 |  |  |  |  |
| 629 | A0A2I2GC58 | 2.45 |  |  |  |  |
| 630 | A0A0U5GTE1 | 4.64 |  |  |  |  |
| 631 | A0A2I2GIW6 | 7.93 |  |  |  |  |
| 632 | P60204 | 11.41 |  |  |  |  |
| 633 | Q0CC87 | 19.37 |  |  |  |  |
| 634 | A0A0S7E3Y9 | 6.45 |  |  |  |  |
| 635 | Q0CC58 | 13.92 |  |  |  |  |
| 636 | Q0CC59 | 36.79 |  |  |  |  |
| 637 | Q0CBS1 | 7.08 |  |  |  |  |
| 638 | Q0CBR4 | 42.45 |  |  |  |  |
| 639 | Q0CC31 | 7.49 |  |  |  |  |
| 640 | Q2U4K2 | 21.13 |  |  |  |  |
| 641 | A0A0F0IGZ4 | 17.46 |  |  |  |  |
| 642 | A0A0F8V3T3 | 26.53 |  |  |  |  |
| 643 | A0A0F8VCN1 | 17.19 |  |  |  |  |
| 644 | A0A1S9DFU7 | 25 |  |  |  |  |
| 645 | A0A100IMS3 | 2.21 |  |  |  |  |
| 646 | A0A2I1D1F1 | 2.99 |  |  |  |  |
| 647 | Q0CAK5 | 36.56 |  |  |  |  |
| 648 | Q0CAJ5 | 14.89 |  |  |  |  |
| 649 | A0A370THZ7 | 27.27 |  |  |  |  |
| 650 | A0A0F0IJT2 | 4.06 |  |  |  |  |
| 651 | Q0CB50 | 24.76 |  |  |  |  |
| 652 | Q0CAT1 | 1.99 |  |  |  |  |
| 653 | A0A017S8A8 | 2.86 |  |  |  |  |
| 654 | Q0CB11 | 42.47 |  |  |  |  |
| 655 | A0A1E3BMT6 | 10.63 |  |  |  |  |
| 656 | A0A0F0I5V0 | 8.27 |  |  |  |  |
| 657 | A0A397GBQ7 | 8.4 |  |  |  |  |
| 658 | A0A254TU92 | 2.79 |  |  |  |  |
| 659 | A0A2V5H1K9 | 4.69 |  |  |  |  |
| 660 | Q0CAY8 | 12.92 |  |  |  |  |
| 661 | Q0CAX5 | 8.33 |  |  |  |  |
| 662 | A0A0F8V4T4 | 7.28 |  |  |  |  |
| 663 | A0A0F0IAN9 | 8.13 |  |  |  |  |
| 664 | A0A100ILI8 | 6.65 |  |  |  |  |
| 665 | A0A017SD54 | 5.43 |  |  |  |  |
| 666 | G3K507 | 2.94 |  |  |  |  |
| 667 | A0A017SSW8 | 3.81 |  |  |  |  |
| 668 | Q0CBD4 | 2.88 |  |  |  |  |
| 669 | Q0CBD1 | 44.29 |  |  |  |  |
| 670 | A0A0F0IP46 | 1.66 |  |  |  |  |
| 671 | A0A017S635 | 7 |  |  |  |  |
| 672 | Q2U4K2 | 23.24 |  |  |  |  |
| 673 | Q9HGY8 | 5.98 |  |  |  |  |
| 674 | A0A0F8U541 | 2.96 |  |  |  |  |
| 675 | A0A017S9F7 | 3.53 |  |  |  |  |
| 676 | Q0CA57 | 14.06 |  |  |  |  |
| 677 | Q0CA42 | 5.64 |  |  |  |  |
| 678 | A0A0F8WFP2 | 8.09 |  |  |  |  |
| 679 | A0A2I2G578 | 11.7 |  |  |  |  |
| 680 | Q0CA05 | 15.43 |  |  |  |  |
| 681 | A0A0F8U0N7 | 8.7 |  |  |  |  |
| 682 | A0A1S9DTN5 | 5.21 |  |  |  |  |
| 683 | A0A0J5PYI6 | 7.51 |  |  |  |  |
| 684 | A0A1L9RL98 | 2.28 |  |  |  |  |
| 685 | A0A0F0IB15 | 9.76 |  |  |  |  |
| 686 | A0A017SDP4 | 2.99 |  |  |  |  |
| 687 | Q0CA77 | 19.71 |  |  |  |  |
| 688 | Q0CA78 | 0.71 |  |  |  |  |
| 689 | Q0CAE1 | 7.83 |  |  |  |  |
| 690 | P85978 | 5.84 |  |  |  |  |
| 691 | A0A1L9UG37 | 6.09 |  |  |  |  |
| 692 | A0A370TPX9 | 1.9 |  |  |  |  |
| 693 | A0A017SPG0 | 1.32 |  |  |  |  |
| 694 | Q0CGV7 | 28.39 |  |  |  |  |
| 695 | Q0CH74 | 22.57 |  |  |  |  |
| 696 | Q0CGV1 | 1.98 |  |  |  |  |
| 697 | Q0CH63 | 13.7 |  |  |  |  |
| 698 | Q0CH50 | 14.6 |  |  |  |  |
| 699 | Q0CGR7 | 4.31 |  |  |  |  |
| 700 | Q0CGR6 | 13.64 |  |  |  |  |
| 701 | A0A0K8L110 | 15 |  |  |  |  |
| 702 | A0A017SQX4 | 3.26 |  |  |  |  |
| 703 | A0A0K8LFW1 | 2.85 |  |  |  |  |
| 704 | A0A017SMB1 | 3.23 |  |  |  |  |
| 705 | Q0CHK5 | 6.22 |  |  |  |  |
| 706 | A0A0F8TZ60 | 10.45 |  |  |  |  |
| 707 | A0A0K8LJN1 | 7.59 |  |  |  |  |
| 708 | Q0CHI4 | 16.12 |  |  |  |  |
| 709 | A0A0F0I8N7 | 9.94 |  |  |  |  |
| 710 | A0A017S473 | 6.61 |  |  |  |  |
| 711 | A0A2I1CX77 | 20.77 |  |  |  |  |
| 712 | Q0CI14 | 58.7 |  |  |  |  |
| 713 | A0A017SQZ4 | 3.8 |  |  |  |  |
| 714 | A0A0F8V271 | 6.12 |  |  |  |  |
| 715 | A0A017S964 | 9.13 |  |  |  |  |
| 716 | A0A318ZJV4 | 2.06 |  |  |  |  |
| 717 | A0A146EYW8 | 2.74 |  |  |  |  |
| 718 | A0A1M3SZ39 | 5.66 |  |  |  |  |
| 719 | Q0CFS0 | 4.13 |  |  |  |  |
| 720 | Q0CFQ7 | 39.3 |  |  |  |  |
| 721 | Q0CG22 | 29.29 |  |  |  |  |
| 722 | A0A0J5Q197 | 3.12 |  |  |  |  |
| 723 | Q0CG18 | 8.65 |  |  |  |  |
| 724 | Q0CG10 | 9.51 |  |  |  |  |
| 725 | A0A0J5PPQ1 | 6.8 |  |  |  |  |
| 726 | A2RQB8 | 8.03 |  |  |  |  |
| 727 | Q9P443 | 5.06 |  |  |  |  |
| 728 | Q0CGG8 | 16.83 |  |  |  |  |
| 729 | A0A017S9F0 | 8.46 |  |  |  |  |
| 730 | A0A100INB6 | 11.96 |  |  |  |  |
| 731 | Q0CGC5 | 21.76 |  |  |  |  |
| 732 | A0A0F8UXZ6 | 3.11 |  |  |  |  |
| 733 | C8VAU8 | 2.43 |  |  |  |  |
| 734 | A0A0F0I698 | 4.08 |  |  |  |  |
| 735 | Q0CGL5 | 46.71 |  |  |  |  |
| 736 | A0A0F0IBT5 | 2.7 |  |  |  |  |
| 737 | A0A017SBM4 | 2.16 |  |  |  |  |
| 738 | A0A0F0I900 | 4.44 |  |  |  |  |
| 739 | A0A017SCW9 | 4.29 |  |  |  |  |
| 740 | A0A1L9UQK8 | 3.01 |  |  |  |  |
| 741 | Q0CF36 | 12.84 |  |  |  |  |
| 742 | Q0CER7 | 13.8 |  |  |  |  |
| 743 | Q0CF08 | 25.69 |  |  |  |  |
| 744 | Q0CEN8 | 5.53 |  |  |  |  |
| 745 | A0A017S7P5 | 6.02 |  |  |  |  |
| 746 | Q0CEZ1 | 20.13 |  |  |  |  |
| 747 | A0A017SC42 | 2.89 |  |  |  |  |
| 748 | Q0CEX9 | 3.94 |  |  |  |  |
| 749 | Q0CF90 | 17.86 |  |  |  |  |
| 750 | A0A0E3JLC0 | 5.64 |  |  |  |  |
| 751 | Q0CFG2 | 31.05 |  |  |  |  |
| 752 | A0A0F0I2Z7 | 12.99 |  |  |  |  |
| 753 | A0A100ILS4 | 4.26 |  |  |  |  |
| 754 | Q0CFC5 | 30.4 |  |  |  |  |
| 755 | Q0CFC6 | 6.86 |  |  |  |  |
| 756 | Q0CFC3 | 20.26 |  |  |  |  |
| 757 | Q0CFC4 | 15.5 |  |  |  |  |
| 758 | A0A100IBW2 | 4.23 |  |  |  |  |
| 759 | Q0CFB9 | 9.75 |  |  |  |  |
| 760 | A1DKA5 | 1.37 |  |  |  |  |
| 761 | Q0CFK0 | 20.17 |  |  |  |  |
| 762 | Q0CE43 | 11.32 |  |  |  |  |
| 763 | P85978 | 7.14 |  |  |  |  |
| 764 | Q0CE38 | 2.63 |  |  |  |  |
| 765 | A0A0U5FTH9 | 2.53 |  |  |  |  |
| 766 | Q0C9S9 | 22.14 |  |  |  |  |
| 767 | A0A0J5PUM3 | 6.69 |  |  |  |  |
| 768 | A0A1E3BRH1 | 11.04 |  |  |  |  |
| 769 | Q0CE18 | 49.67 |  |  |  |  |
| 770 | P38091 | 12.39 |  |  |  |  |
| 771 | Q0CDY0 | 15.66 |  |  |  |  |
| 772 | Q0CE87 | 2.26 |  |  |  |  |
| 773 | Q0CDW4 | 2.98 |  |  |  |  |
| 774 | Q0CDV8 | 1.93 |  |  |  |  |
| 775 | A0A2I2GH26 | 4.87 |  |  |  |  |
| 776 | A0A0F8VKQ5 | 5.61 |  |  |  |  |
| 777 | Q0CDT5 | 8.67 |  |  |  |  |
| 778 | A0A0F0I9E1 | 13.42 |  |  |  |  |
| 779 | Q0CED1 | 11.29 |  |  |  |  |
| 780 | Q1HFV4 | 1.14 |  |  |  |  |
| 781 | A0A017SGE2 | 5.5 |  |  |  |  |
| 782 | A0A1L9V1A4 | 20.29 |  |  |  |  |
| 783 | Q0CEJ2 | 4.23 |  |  |  |  |
| 784 | A0A0J5Q3R6 | 7.11 |  |  |  |  |
| 785 | Q0CEI5 | 2.81 |  |  |  |  |
| 786 | A0A0S7DS48 | 2.86 |  |  |  |  |
| 787 | A0A1S9DXV2 | 2.33 |  |  |  |  |
| 788 | Q0CEF4 | 17.67 |  |  |  |  |
| 789 | A0A017SHP9 | 19.51 |  |  |  |  |
